# *In vitro* and *in silico* evaluations of actinomycin X_2_and actinomycin D as potent anti-tuberculosis agents

**DOI:** 10.7717/peerj.14502

**Published:** 2023-03-08

**Authors:** Kamal Ahmad Qureshi, Faizul Azam, Muhammad Qaiser Fatmi, Mahrukh Imtiaz, Dinesh Kumar Prajapati, Pankaj Kumar Rai, Mariusz Jaremko, Abdul-Hamid Emwas, Gamal Osman Elhassan

**Affiliations:** 1 Department of Pharmaceutics, Unaizah College of Pharmacy, Qassim University, Unaizah, Al-Qassim, Saudi Arabia; 2 Department of Pharmaceutical Chemistry and Pharmacognosy, Unaizah College of Pharmacy, Qassim University, Unaizah, Al-Qassim, Saudi Arabia; 3 Department of Biosciences, COMSATS University Islamabad, Islamabad, Pakistan; 4 Department of Biotechnology, Faculty of Biosciences, Invertis University, Bareilly, Uttar Pradesh, India; 5 Smart-Health Initiative (SHI) and Red Sea Research Center (RSRC), Division of Biological and Environmental Sciences and Engineering (BESE), King Abdullah University of Science and Technology (KAUST), Thuwal, Jeddah, Saudi Arabia; 6 Core Labs, King Abdullah University of Science and Technology (KAUST), Thuwal, Saudi Arabia

**Keywords:** Actinomycin, Streptomyces, Anti-TB drug, Molecular docking, Protein kinase PknB

## Abstract

**Background:**

Multidrug-resistant tuberculosis (MDR-TB) is one of the world’s most devastating contagious diseases and is caused by the MDR-*Mycobacterium tuberculosis* (MDR-Mtb) bacteria. It is therefore essential to identify novel anti-TB drug candidates and target proteins to treat MDR-TB. Here, *in vitro* and *in silico* studies were used to investigate the anti-TB potential of two newly sourced actinomycins, actinomycin-X_2_ (act-X_2_) and actinomycin-D (act-D), from the *Streptomyces smyrnaeus* strain UKAQ_23 (isolated from the Jubail industrial city of Saudi Arabia).

**Methods:**

The anti-TB activity of the isolated actinomycins was assessed *in vitro* using the Mtb H37Ra, *Mycobacterium bovis* (BCG), and Mtb H37Rv bacterial strains, using the Microplate Alamar Blue Assay (MABA) method. *In silico* molecular docking studies were conducted using sixteen anti-TB drug target proteins using the AutoDock Vina 1.1.2 tool. The molecular dynamics (MD) simulations for both actinomycins were then performed with the most suitable target proteins, using the GROningen MAchine For Chemical Simulations (GROMACS) simulation software (GROMACS 2020.4), with the Chemistry at HARvard Macromolecular Mechanics 36m (CHARMM36m) forcefield for proteins and the CHARMM General Force Field (CGenFF) for ligands.

**Results:**

*In vitro* results for the Mtb H37Ra, BCG, and Mtb H37Rv strains showed that act-X_2_ had minimum inhibitory concentration (MIC) values of 1.56 ± 0.0, 1.56 ± 0.0, and 2.64 ± 0.07 µg/mL and act-D had MIC values of 1.56 ± 0.0, 1.56 ± 0.0, and 1.80 ± 0.24 µg/mL respectively. The *in silico* molecular docking results showed that protein kinase PknB was the preferred target for both actinomycins, while KasA and pantothenate synthetase were the least preferred targets for act-X_2_and act-D respectively. The molecular dynamics (MD) results demonstrated that act-X_2_ and act-D remained stable inside the binding region of PknB throughout the simulation period. The MM/GBSA (Molecular Mechanics/Generalized Born Surface Area) binding energy calculations showed that act-X_2_ was more potent than act-D.

**Conclusion:**

In conclusion, our results suggest that both actinomycins X_2_ and D are highly potent anti-TB drug candidates. We show that act-X_2_is better able to antagonistically interact with the protein kinase PknB target than act-D, and thus has more potential as a new anti-TB drug candidate.

## Introduction

Tuberculosis (TB) is a highly contagious disease, caused by the *Mycobacterium tuberculosis* (Mtb) bacteria. It is spread by airborne transmission when an infected person coughs and spits the bacteria into the air. The infection can be caught at any age, and mainly affects the lungs; although it can affect any organ (“Tuberculosis”). In 2019 alone, the World Health Organisation (WHO) reported 1.4 million deaths due to TB. TB is one of the top ten causes of death globally and the leading cause of death by a single infectious disease, surpassing HIV/AIDS (“Tuberculosis”; Chen et al., 2012; Müller, 2016; Jiang et al., 2021). TB affects approximately a third of the world’s population, with a new infection emerging at a rate equivalent to one person every second (Chen et al., 2012).

TB can be treated by taking various medications for 6–9 months. The United States Food and Drug Administration (USFDA) has approved ten drugs to treat TB (“Tuberculosis”; Müller, 2016); these include rifampin (RIF), isoniazid (INH), pyrazinamide (PZA), and ethambutol (EMB), which are considered first-line anti-TB medicines, and provide the foundation for most treatment regimens (“Tuberculosis”; Müller, 2016).

Multidrug-resistant tuberculosis’ (MDR-TB) (MDR-TB) is caused when Mtb is resistant to more than one anti-TB drug, and at least INH and RIF (“Tuberculosis”; Müller, 2016). MDR-TB is a global health emergency and a threat to public safety. Approximately 200,000 people worldwide have been identified as having multi-drug- or rifampicin-resistant tuberculosis (MDR/RR-TB) (“Tuberculosis”; Müller, 2016). Treating MDR-TB is challenging—with a potential for life-threatening consequences, if managed inappropriately. The number of suitable drugs available is limited, and drug resistance is a major issue; new therapeutic targets and drugs able to treat MDR-TB are therefore needed. The actinomycetes genus, *Streptomyces,* produces many important bioactive molecules with economic and medical benefits, including: antibiotics, antitumor, antiviral, herbicidal, and insecticidal agents. Consequently, this genus and its constituents are gathering more attention (Tanaka & Omura, 1993; Singh, Baruah & Bora, 2006; Sanasam & Ningthoujam, 2010; Chen et al., 2012; Qureshi et al., 2021a; Qureshi et al., 2021b)

Actinomycins are chromopeptide lactone antibiotics, derived from several *Streptomyces* strains, predominantly exhibiting anti-cancer and anti-microbial properties (Tanaka & Omura, 1993; Singh, Baruah & Bora, 2006; Sanasam & Ningthoujam, 2010; Chen et al., 2012). Act-D is widely utilized in clinical practice to treat various tumors, particularly Wilms tumors and pediatric rhabdomyosarcomas (Chen et al., 2012). Act-D has become an essential tool in medical, developmental and molecular genetics as it is able to crosslink into duplex DNA, inhibit DNA-dependent RNA polymerase, and inhibit protein synthesis (Chen et al., 2012). In one study, act-D suppressed the coxsackievirus B3 and was proposed to treat AIDS (acquired immunodeficiency syndrome) due to its ability to block the minus-strand transfer mechanism in HIV-1 (Human Immunodeficiency Virus-1) (Chen et al., 2012).

Many *Streptomyces* strains produce act-X_2_ and act-D; and although these actinomycins exhibit substantial anti-TB activity (Praveen & Tripathi, 2009; Zhang et al., 2009; Chen et al., 2012; Chen et al., 2020; Wang et al., 2017a; Wei et al., 2017; Ali et al., 2018; Sharma & Manhas, 2019; Jamal, 2020), there are no molecular docking or dynamics studies to support their anti-TB activity.

We previously isolated, identified, and characterized a novel actinomycete strain, known as *S. smyrnaeus* strain UKAQ_23 (Fig. S1), which was obtained from a mangrove sediment sample in the Jubail industrial city of Saudi Arabia. We also produced, purified, identified, and characterized actinomycins X_2_ and D, which are synthesized by the *S. smyrneus* strain UKAQ_23 (Figs. S2–S3) (Qureshi et al., 2021a). This current study is an extension of our previous work.

Here, we investigate the anti-TB potential of actinomycins X_2_ and D (produced by *S. smyrnaeus* strain UKAQ_23) through *in vitro* and *in silico* studies, using 16 known anti-TB drug targets. Also, we show the mode of action for the anti-TB activity of act-X_2_ and act-D against Mtb. This is the first study using molecular docking and dynamics studies to demonstrate the anti-TB potential of act-X_2_ and act-D.

## Materials & Methods

### Test organisms

The Mtb H37Ra, BCG, and Mtb H37Rv were used as test organisms.

### Isolation and identification of *S. smyrnaeus* strain UKAQ_23

The novel *S. smyrnaeus* strain UKAQ_23 was isolated from a mangrove sediment sample collected from the Jubail industrial city of Saudi Arabia (Fig. S1). The strain UKAQ_23 was identified as a novel *S. smyrnaeus* strain UKAQ_23 by 16S rRNA gene sequencing. The 16S rRNA gene sequences of UKAQ_23 were submitted to GenBank (NCBI) with accession number MG657032.1. A culture copy of *S. smyrnaeus* strain UKAQ_23 has also been deposited at the National Centre for Microbial Resource (NCMR), Pune, MS, India, under the accession number MCC 0192. Our previous publication provides detailed information on this novel strain UKAQ_23 (Qureshi et al., 2021a).

### Production, extraction, purification, and identification of actinomycins X_2_ and D

The novel strain UKAQ_23 was used to produce actinomycins using a modified ISP-4 fermentation medium and the solid-state fermentation method (Fig. S2). The actinomycins were extracted, purified, and identified as act-X_2_ and act-D. Our previous publication provides more detailed information on act-X_2_ and act-D (Qureshi et al., 2021b; Qureshi et al., 2021a).

### *In vitro* Anti-TB activity of act-X_2_ and act-D

The *in vitro* anti-TB activity of act-X_2_ and act-D was conducted using the Microplate Alamar Blue Assay (MABA) method (Collins & Franzblau, 1997; Franzblau et al., 1998; Amin et al., 2008; Mendoza-Aguilar et al., 2012; Cho, Lee & Franzblau, 2015). The minimum inhibitory concentration (MIC) values for act-X_2_ and act-D against Mtb H37Ra and BCG were determined using a single read-out assay (fluorescence), while the MIC values for act-X_2_ and act-D against Mtb H37Rv were calculated using a dual read-out assay (optical density and fluorescence). The mean of the two MIC values (from both single and dual read-out assays) was used. Each test was performed in triplicate. The results are expressed using the mean ± standard deviations (SD).

Rifampicin (2 µg/mL), streptomycin (6 µg/mL), rifampin (0.02 µg/mL), isoniazid (0.46 µg/mL), linezolid (0.92 µg/mL), and moxifloxacin (0.19 µg/mL) were used as positive control drugs.

### Microplate Alamar Blue Assay (MABA) cell stock preparation

All solutions and media were sterilized and stored at 4 °C away from direct sunlight until use. The following materials were used in the preparation of MABA cell stocks: sterile 20% (v/v) tween 80, 50% (v/v) glycerol, Middlebrook 7H11 agar medium, Middlebrook 7H9 broth medium, phosphate buffer saline (PBS), PBS-Tween 80 (PBST), tryptic soy agar with 5% sheep blood. All procedures were carried out in a sterile environment. The inocula for the test organism’s seed stocks were prepared by suspending colonies from 7H11 agar in 200 mL of 7H9 broth in a 500 mL Nephelo flask, and allowing them to grow until the log phase (40–60 Klett units) was attained; after which they were removed from the flask.  The suspensions were then transferred to 50 mL conical centrifuge tubes and centrifuged at 4,000 × g for 10 min at 4 °C. After centrifugation, the supernatants were discarded, and one mL of sterile 1 x PBS was dispensed into each of 50 mL conical centrifuge tubes, followed by resuspension of pellets by pipetting. Each 50 mL tube was then filled with 30 mL of PBS, and pellets were resuspended in PBS by vortexing for 5 s. All tubes were centrifuged at 4,000 × g for 10 min at 4 °C. After centrifugation, the supernatants were discarded, and each 50 mL conical centrifuge tube was filled with one mL of PBS, followed by resuspension of pellets by pipetting. Aliquots containing 200 µL of suspended cells were transferred into sterile screwcap micro-tubes with a capacity of 1.5 mL. The 200 µL sample was then used to validate sterility on the blood agar plate and determine colony-forming units (CFU) on 6-well plates, following the serial dilution of 10^−4^–10^−7^. All tubes were labeled and stored at −80 °C in a storage box. The Klett units, tube numbers, sterility, CFU test results, date, and location in the freezer (−80 °C) were all recorded on a log sheet. The seed stocks were preserved for up to one year.

### MABA protocol

The following materials were used in the MABA protocol: 20% (v/v) Tween 80, 7H12 medium, quality control (QC) standard anti-TB drugs, *i.e.,* rifampin (0.02 µg/mL), isoniazid (0.46 µg/mL), linezolid (0.92 µg/mL), moxifloxacin (0.19 µg/mL), rifampicin (2 µg/mL), and streptomycin (6 µg/mL), sterile 96-well clear plates, a sonicator, blood agar plate, 6-well plates with five mL of 7H11 agar in each well, Alamar Blue^®^, and a microplate fluorometer with 530 nm excitation and 590 nm emission filters. To prevent medium evaporation from the test wells during incubation, 200 µL of 7H12 medium was dispensed into the outer-perimeter wells of the 96-well plates. All wells in column 3 were filled with 200 µL of 7H12 medium, while all other wells were filled with 100 µL of 7H12 medium. The 2 µL of the listed QC drugs or test actinomycins, X_2_ and D, were added in columns 1–3, rows B-F in a 96-well microtiter plate. Two-fold serial dilutions of act-X_2_ and act-D were then prepared from columns 3 to 10, and at the end of dilution, 100 µL was discarded from column 10. The frozen cell stocks were thawed and sonicated briefly for 3–5 s at 20% power to achieve an even suspension. The cultures were diluted with the 7H12 medium until they reached a density between 5 × 10^4^ and 1 × 10^5^ CFU/mL. Then, 100 µL of culture was dispensed into wells from columns 2–11, rows B–G. The plates were incubated for seven days at 37 °C with 5% CO_2_ and 95% humidity. The sterility of the cell stock was determined by streaking it onto a blood agar plate and incubating it at 37 °C for one week. Ten-fold serial dilutions were prepared up to 10^−7^, and 50 µL of each dilution was then inoculated into 6-well 7H11 agar plates and incubated for three weeks at 37 °C in a CO_2_ incubator. If bacterial or fungal colonies were isolated on the blood agar or 7H11 plates within a week, the batch of cell stock was autoclaved and discarded. After three weeks, the CFU on the 7H11 agar plates was counted and reported. The 96-well plates were removed from the incubator on the seventh day of incubation. The Alamar Blue-Tween 80 solution was prepared by diluting Alamar Blue in an 8:5 ratio with 20% (v/v) Tween 80 (for one plate, 2 mL Alamar Blue and 1.25 mL 20% (v/v) Tween 80 solution were required). A 32.5 µL of Alamar Blue-Tween 80 was added to each well in the 96-well plates. Plates were incubated for 18–24 h at 37 °C, 5% CO_2_, and 95% humidity. The fluorescence was measured at 530 nm excitation and 590 nm emission. The MIC_90_ was then determined using the following formula:

(1) \begin{eqnarray*}90\text{%}~\text{inhibition}~({\text{MIC}}_{90})=0.1\times (\text{Bacterial Control}-\text{Background}).\end{eqnarray*}

The MIC_90_ value is defined as the lowest concentration of a test compound that inhibits fluorescence by 90% compared to an untreated bacterial control.

### Statistical analysis

The significance of the differences in the mean MIC_90_ values of act-X_2_ and act-D were determined using a one-way ANOVA, with a significance level of *p* = 0.05. Each test was performed in triplicate. The statistical analyses were conducted using SPSS software, version 20.0 (IBM Corp., Armonk, NY, USA) (Qureshi et al., 2022).

### *In silico* molecular docking studies

X-ray crystal structures of anti-TB drug targets (protein kinase PknB, polyketide synthase-13, lumazine synthase, pantothenate kinase, decaprenylphosphoryl- *β*-D-ribose-2′-oxidase (DprE1), protein tyrosine phosphatase PtpB, DNA gyrase B-ATPase domain, enoyl reductase, DNA topoisomerase-I, DNA ligase, *β*-ketoacyl synthase-A, mycobacterium type II dehydroquinase, diacylglycerol O-acyltransferase, mycobacterium shikimate kinase, and pantothenate synthetase) were obtained from the Research Collaboratory for Structural Bioinformatics Protein Data Bank (RCSB PDB, http://www.rcsb.org/pdb/home/home.do) with PDB IDs 1MNV, 2FUM, 5V3X, 2C92, 4BFT, 6HEZ, 2OZ5, 4B6C, 4U0J, 5D5H, 6 kJM, 2WGE, 2Y71, 5KWI, 2IYQ, and 3IVX, respectively (Table S1).

Each protein was handled individually in the Biovia Discovery Studio Visualizer 2020 and MGLTools 1.5.6 programs, and standard procedures were followed for the preparation of receptors—including deletion of co-crystallized ligands, water molecules, and cofactors and addition of Gasteiger charges (Shushni, Azam & Lindequist, 2013; Hussain et al., 2020). Three-dimensional structures of the compounds act-X_2_ and act-D were retrieved from the PubChem database. The ligand preparation module of MGL Tools 1.5.6 was used for merging all non-polar hydrogens, and to define rotatable bonds of the ligands.

AutoDock Vina 1.1.2 was used for molecular docking analysis using the default protocol (Trott & Olson, 2009). A grid box was built with a grid spacing of 1 Å at the center of respective co-crystallized ligands, with dimensions of 30 points in all directions for each receptor. Upon completing the docking, the best poses were selected from the top ten models from each target by examining their binding energy (ΔG_binding_, kcal/mol) and non-bonded interactions profile (Azam, Eid & Almutairi, 2021). Biovia Discovery Studio Visualizer 2020 and PyMol 1.7.4 were used for studying molecular interactions.

### *In silico* molecular dynamics (MD) simulations

After the detailed docking analysis, the best complexes with lowest binding energies were selected. MD simulations of Protein Kinase PknB complexed with act-D and act-X_2_ were then performed for 250 ns using the GROningen MAchine For Chemical Simulations (GROMACS) simulation software (GROMACS 2020.4) (Abraham et al., 2015), using the Chemistry at HARvard Macromolecular Mechanics 36m (CHARMM36m) (Huang et al., 2016) forcefield for proteins and the CHARMM General Force Field (CGenFF) for act-X_2_ and act-D. In a truncated octahedral box, both protein-ligand systems were solvated with TIP3P water molecules. The minimum distance between protein systems and the edges of the simulation box was set to 10 Å, to efficiently meet the criteria for minimum image convention during MD simulation. The 0.15M KCl ionic concentration was introduced by adding 39 K^+^ and 33 Cl^−^ ions to the environment. The protonation states for His, Tyr, Lys, Arg, Asp, and Glu residues were evaluated at pH 7 and 0.15M of salinity using an H++ web server (version 3.2) and implemented after visual inspection (Anandakrishnan, Aguilar & Onufriev, 2012). The systems for act-D and act-X_2_ contained a total of 39,707 and 39,787 atoms, respectively. The Chemistry at HARvard Macromolecular Mechanics Graphical User Interface (CHARMM-GUI) webserver was used to generate all input files (Jo et al., 2008; Lee et al., 2016).

Both systems were minimized for 5,000 steps using the Steepest Descent technique. Convergence was achieved under the force limit of 1,000 (kJ/mol/nm) to exclude any steric clashes. Later, both minimized systems were separately equilibrated at NVT (Canonical ensemble: where moles, N; volume, V; and temperature, T were conserved) and NPT (Isothermal-Isobaric ensemble: where moles, N; pressure, P; and temperature, T were conserved) ensembles for 100 ps (50,000 steps) and 1000 ps (1,000,000 steps), respectively, using time steps 0.2 and 0.1 fs, at 300 K to ensure a fully converged system for the production run (Qureshi et al., 2021a; Qureshi et al., 2021b).

The simulation runs for both systems were conducted at a constant temperature of 300 K and a pressure of 1 atm, or 1 bar (using an NPT ensemble), utilizing weak coupling velocity re-scaling (modified Berendsen thermostat) and Parrinello-Rahman algorithms, respectively. The relaxation times were set at *τ T* = 0.1 ps and *τ P* = 2.0 ps. Using the LINear Constraint Solver (LINCS) algorithm, all bond lengths involving hydrogen atoms were maintained stiffly at optimal bond lengths, with a time step of 2 fs. The non-bonded interactions were calculated using Van der Waals and Coulomb potentials. Interactions within a short-range cutoff of 12 Åwere calculated in each time step. The electrostatic interactions and forces in a homogeneous medium beyond the short-range cutoff were calculated using the Particle Mesh Ewald (PME) method. The Periodic Boundary Conditions (PBC) were applied in all *x*, *y*, and *z* directions. The production was run for 250 ns for each of the three complexes. The trajectory and energy data were recorded at every 10 ps (Qureshi et al., 2021b). The first 25 ns of the trajectories were excluded for comprehensive analysis, and the remaining 225 ns were utilized (except for MM/GBSA calculations, where the first 50 ns of the trajectories were excluded, and the remaining 200 ns were utilized). GRaphing, Advanced Computation and Exploration of data (Grace) was used to generate all plots (https://plasma-gate.weizmann.ac.il/Grace). The Molecular Mechanics/Generalized-Born Surface Area (MM/GBSA) protein-ligand binding energy was averaged over 200 frames calculated at the interval of 1ns over the last 200 ns of 250 ns production run (Miller et al., 2012). The following formula was used to calculate the binding free energy:

(2) \begin{eqnarray*}\Delta {G}_{\mathrm{binding}}=\Delta {G}_{\mathrm{complex}}-(\Delta {G}_{\mathrm{protein}}+\Delta {G}_{\mathrm{ligand}}).\end{eqnarray*}

Five additional MD simulations (replicates) for each of the two systems (act-X_2_ and act-D) were also carried out to authenticate the ligand binding further. The starting conformation for each simulation was taken after every 50 ns for a 250 ns long trajectory (*i.e.,* 50 ns, 100 ns, 150 ns, 200 ns and 250 ns). Before the final production run, each system was subjected to energy minimization and equilibration at both NVT and NPT ensembles. Each simulation was run for 50 ns, followed by MM/GBSA calculations. As previously mentioned, all other parameters and values remained the same.

## Results

### *In vitro* anti-TB activity of act-X_2_ and act-D

**Figure 1:**
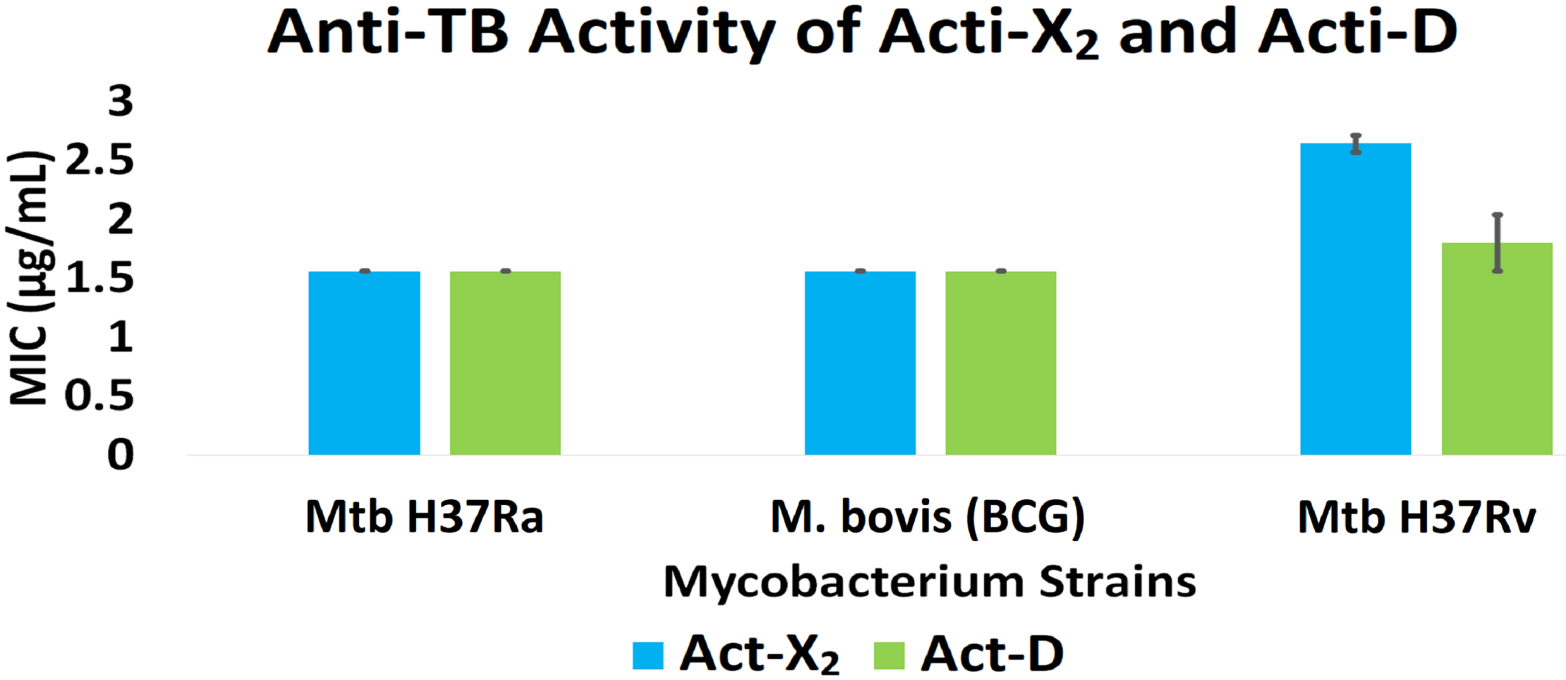
*In vitro* anti-TB activity of isolated act-X_2_ and act-D.

Act-X_2_ and act-D exhibited substantial anti-TB activity against the tested mycobacterial strains. When tested against Mtb H37Ra, BCG, and Mtb H37Rv, act-X_2_ exhibited MIC values of 1.56 ± 0.0, 1.56 ± 0.0, and 2.64 ± 0.07 µg/mL, while act-D showed MIC values of 1.56 ± 0.0, 1.56 ± 0.0, and 1.80 ± 0.24 µg/mL, respectively (Fig. 1 and Table S2). Act-X_2_ and act-D had similar MIC values against the Mtb H37Ra and BCG strains, although, the MIC value was lower when act-D was tested against the Mtb H37Rv strain. This indicates that actinomycins X_2_ and D are more potent against the Mtb H37Ra and BCG strains than the Mtb H37Rv strain.

### Statistical analysis

A statistically significant difference (*p* < 0.05) was observed between the anti-TB activity of act-X_2_ and act-D, as determined by one-way ANOVA (analysis of variance); F (1, 2) = 22.471, *p* = 0.042 (Table S3).

### *In silico* molecular docking studies

To gain insight into the potential interaction modes of act-X_2_ and act-D, a molecular docking study was performed using several anti-TB drug targets (Timo et al., 2019; Baptista et al., 2021). The predicted binding energies of the act-X_2_ and act-D compounds are presented in Table 1.

**Figure 2:**
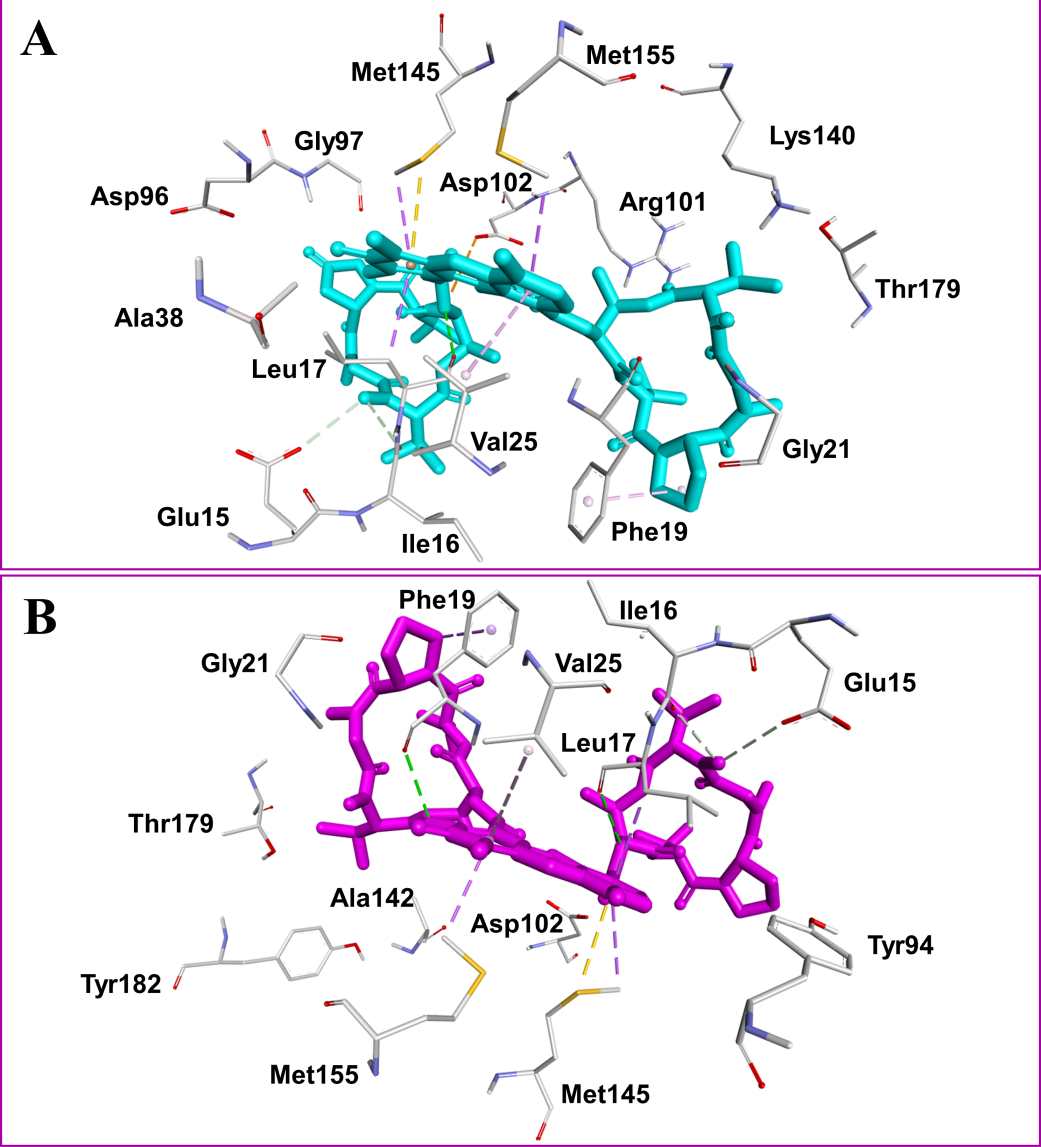
Docked compounds act-X_2_, shown as sticks in cyan color (A); and act-D, shown as sticks in magenta color (B) in the binding pocket of mycobacterial protein kinase PknB. Intermolecular interactions are depicted as broken lines.

**Figure 3:**
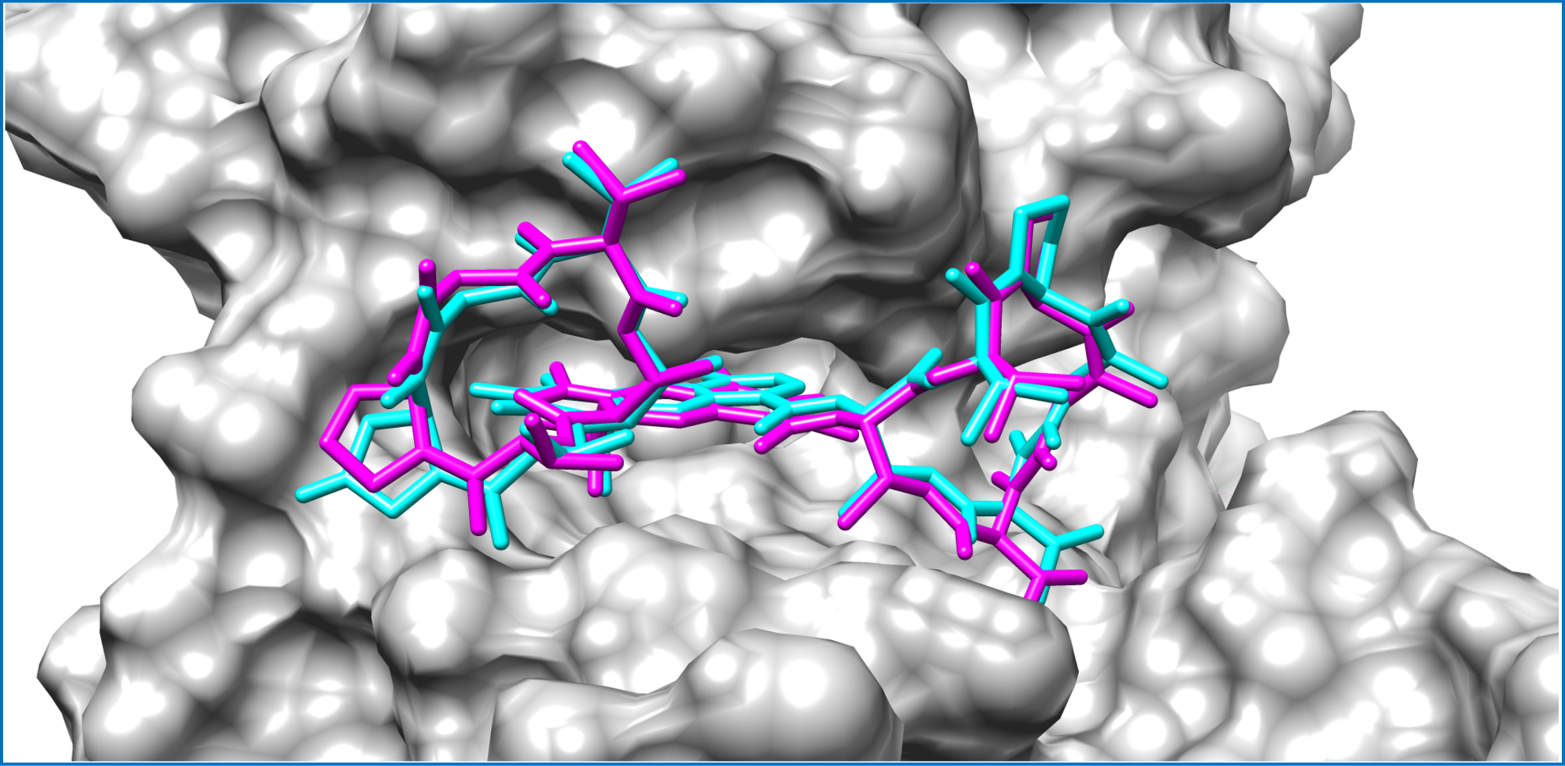
Mycobacterial protein kinase PknB, shown as a surface in a grey color to highlight the binding cavity. Docked compounds act-X_2_ and act-D are shown as sticks in cyan and magenta color, respectively.

Figures 2–3 and Figs. S4–S19 illustrate the binding energies of the docked molecules with the various anti-TB drug targets. The bar plot clearly shows variations in the affinities for the proteins studied. PknB plays a role in multiple mycobacterial biochemical pathways, and is crucial for *in vitro* growth and survival of the microbe in the host (Chawla et al., 2014). Of the 16 drug targets studied, PknB was the most promising target for both act-X_2_ and act-D—with binding energies of −11.7 and −11.8 kcal/mol, respectively. In addition, both compounds, act-X_2_ and act-D preferentially bound to DNA —indicating that they were equipotent in terms of docking, with a predicted binding affinity of −10.6 kcal/mol (Fig. S19).

**Table 1:** Anti-TB drug targets and protein-ligand binding energies obtained from molecular dockings.

**#**	**Targets**	**PDB ID**	**Binding energy (kcal/mol)**	
			**Act-X**_**2**_	**Act-D**
1.	DNA	1MNV	−10.6	−10.6
2.	Protein kinase PknB	2FUM	−11.7	−11.8
3.	Polyketide synthase 13	5V3X	−10.1	−9.6
4.	Lumazine synthase	2C92	−8.8	−9.4
5.	Pantothenate kinase	4BFT	−8.6	−8.5
6.	DprE1	6HEZ	−8.4	−8.4
7.	Protein tyrosine phosphatase PtpB	2OZ5	−7.9	−8.0
8.	DNA GyrB ATPase domain	4B6C	−7.2	−7.4
9.	Enoyl reductase	4U0J	−7.3	−7.4
10.	DNA topoisomerase I	5D5H	−7.2	−7.2
11.	NAD^+^-dependent DNA ligase A	6 kJM	−7.3	−7.0
12.	KasA	2WGE	−5.7	−6.7
13.	Mtb type II dehydroquinase	2Y71	−6.9	−6.6
14.	Diacylglycerol acyltransferase/mycolyltransferase Ag85C	5KWI	−6.4	−6.3
15.	Mtb shikimate kinase	2IYQ	−6.6	−6.2
16.	Pantothenate synthetase	3IVX	−6.1	−6.0

KasA was the least favorable target protein for act-X_2_ (binding energy: −5.7 kcal/mol), and pantothenate synthetase was the least favorable target for act-D (binding energy: −6.0 kcal/mol). Variable affinities were observed for all other targets (Fig. S4 and Table 1).

Actinomycins X_2_ and D are structurally similar, apart from an additional keto group on the proline moiety of act-X_2_. A slight difference in the predicted docking binding energies of both compounds was therefore recorded (Table 1). Act-X_2_ has an additional hydrogen bond acceptor, in the form of a keto moiety, which is an advantage for intermolecular interactions. The intermolecular interactions of the docked compounds act-X_2_ and act-D have been visualized and are presented in Figs. 2–3 and Figs. S5–S19. Mitoxantrone (the native co-crystallized ligand known as 1,4-dihydroxy-5,8-bis({2-[(2-hydroxyethyl)amino]ethyl}amino)anthra-9,10-quinone) is an ATP-competitive inhibitor, encircled by a number of amino acid residues (Leu17, Gly18, Phe19, Val25, Ala38, Glu93, Tyr94, Val95, Gly97, Asp102, Lys140, Ala142, Asn143, Met145, Met155, and Asp156), which collectively form the active site of the mycobacterial protein kinase PknB (Fig. 3) (Wehenkel et al., 2006). The docked compounds, act-X_2_ and act-D, occupy the binding pocket of the PknB protein in a similar manner to mitoxantrone. In the binding site, Glu15, Ile16, Leu17, Phe19, Gly20, and Arg101 formed hydrogen bond interactions with the docked compounds (Fig. 3), whereas hydrophobic contacts were established by the amino acid residues: Leu17, Phe19, Val25, Ala38, Ala142, Met145, and Met155.

### *In silico* MD simulations

**Figure 4:**
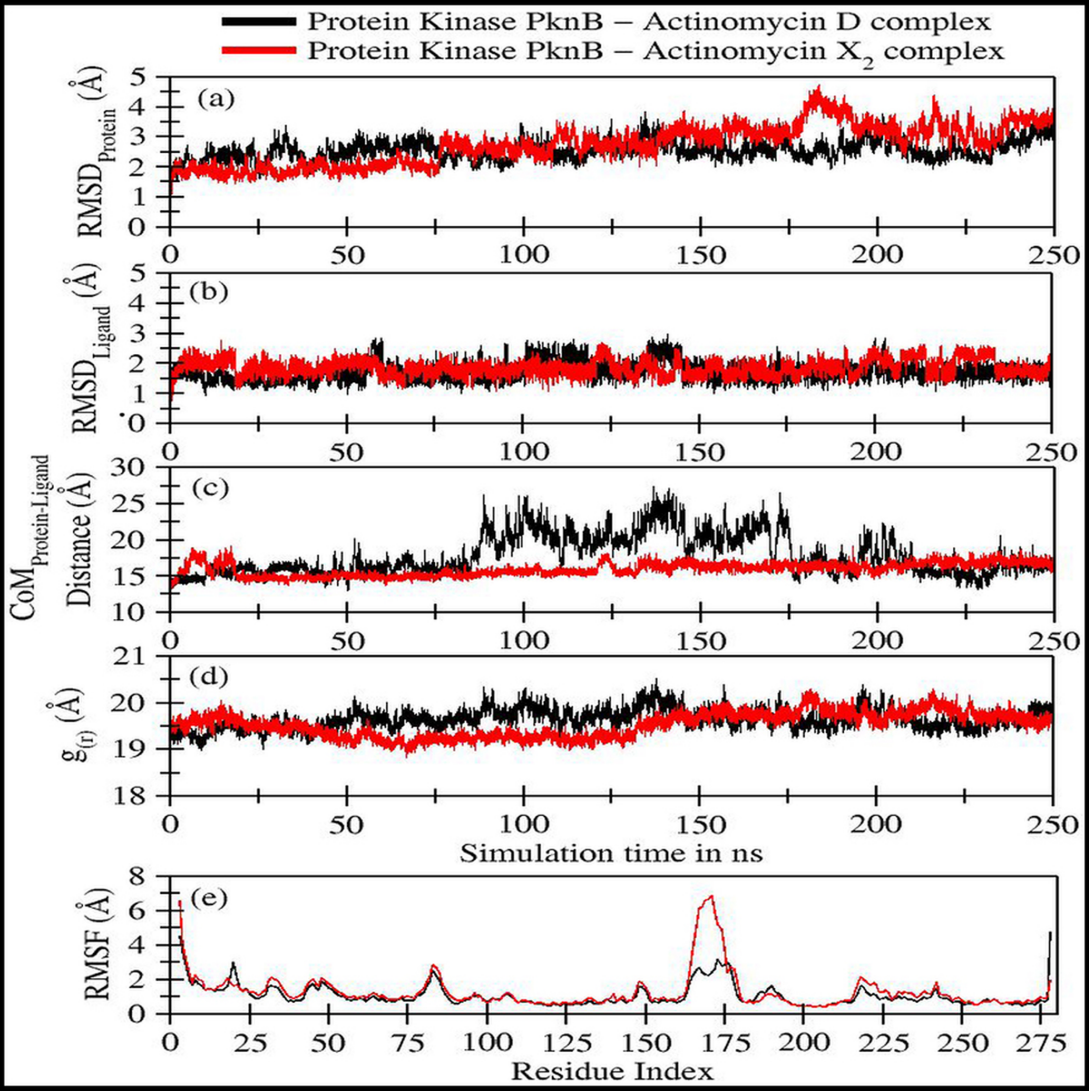
Various trajectory analyses for protein kinase (PknB) complexed with actinomycin D (black lines) and actinomycin X_2_ (red lines): (A) RMSD_protein_, (B) RMSD_ligand_, (C) Center-of-mass distance between protein and ligand, (D) Protein’s radius of gyration, and (E) RMSF. RMSD_protein_, Rg, and RMSF have been calculated using ‘C-alpha’ atoms in the R program using the Bio3D module, while CoM was calculated using gmx distance in Gromacs.

**Figure 5:**
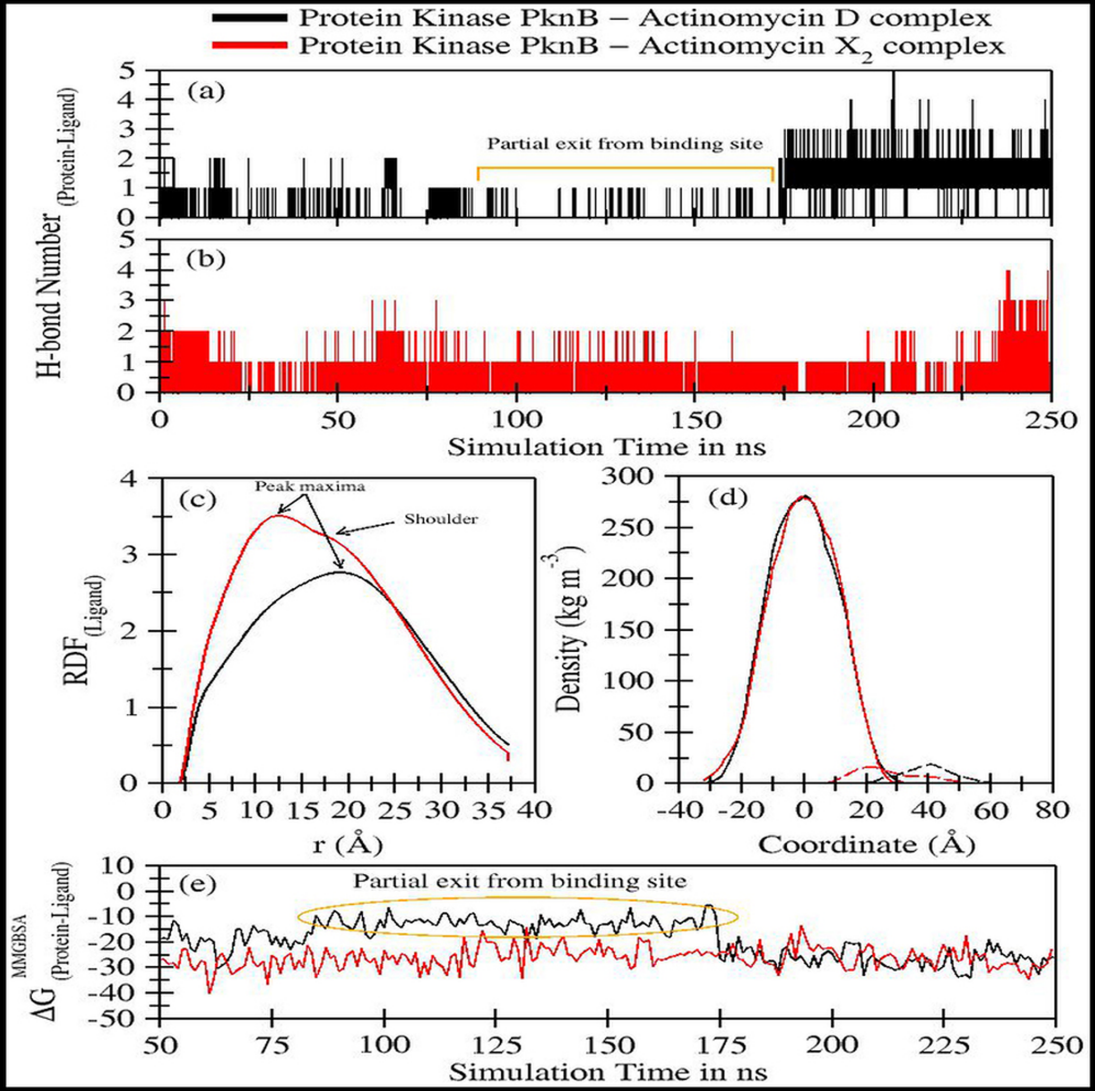
Trajectory analyses for protein kinase (PknB) complexed with act-D (black lines) and act-X_2_ (red lines): (A–B) Number of hydrogen-bonds formed between the protein and the act-D and act-X_2_, (C) Radial distribution functions of act-D and act-X_2_ around PknB, (D) partial density of proteins (solid lines) and ligands (dashed lines), and (E) Molecular Mechanics/Generalized-Born Surface Area protein-ligand binding energy calculated over an interval of 1 ns.

The trajectory analyses for act-X_2_ and act-D in complex with protein kinase PknB were performed in terms of root mean square deviation (RMSD) of protein and ligand, root mean square fluctuations (RMSF), the radius of gyration (g_(r)_), and the center-of-mass distance (CoM) between the protein and ligands (Fig. 4); their mean values are given in Table 2. The number of H-bonds, radial distribution function (RDF) of ligand around protein, the partial density of protein and ligands, and MM/GBSA (Miller et al., 2012) ligand-protein binding energies (Fig. 5) were also plotted. The values for various energy contributions are listed in Table 3.

Figure 4A shows the RMSD plot for protein kinase (PknB) in complex with both actinomycins X_2_ (red line) and D (black line). Both systems remained stable throughout the simulation time; although, the mean RMSD value for act-X_2_ (2.85 ± 0.62 Å) was slightly higher than that of act-D (2.59 ± 0.31 Å). The RMSD value increased for act-X_2_ between 175 to 200 ns, which was associated with the stronger movements of residues 157 to 180 forming a loop. Of note, this loop was absent from the crystal structure due to the higher beta-factor value. Overall, the RMSD values fluctuated within the range of ∼3 Å, indicating that both systems were stable and no major conformational changes were observed, other than the loop mentioned above.

Figure 4B displays the RMSD plot for ligands actinomycins X_2_ (red line) and D (black line). Both actinomycins X_2_ and D remained highly stable within the binding pocket of the PknB protein, and exhibited mean RMSD values of 1.87 ± 0.27 Åand 1.77 ± 0.29 Å, respectively. Although act-X_2_ displayed more conformational changes than act-D, the increase in stability was negligible—as reflected by its smaller standard deviation value.

Figure 4C shows the fluctuation in distance between the center-of-masses (CoM) of protein kinase (PknB) and actinomycins X_2_ (red line) and D (black line) during the simulation time. Although act-X_2_ had higher RMSD values, it remained completely intact with the PknB protein binding site, with a mean CoM distance of 15.96 ± 0.87 Å. Act-D partially exited the binding pocket during ∼80 ns to 175 ns of simulation time, with an overall mean CoM distance of 18.17 ± 2.79 Å, but still remained partially intact with the protein. This partial exit of act-D may explain why the ligand-induced local conformational changes in PknB were not as prominent as with act-X_2_.

Figure 4D depicts the radius of gyration (g_(r)_) plot for PknB in complex with actinomycins X_2_ (red line) and D (black line) during the simulation time. Both protein systems remained highly compact, with mean values of 19.53 ± 0.27 Å and 19.68 ± 0.20 Å. They did not exhibit any significant global ligand-induced conformational changes.

Figure 4E shows the root means square fluctuations for PknB in complex with actinomycins X_2_ (red line) and D (black line). Similar patterns of fluctuation were observed for both protein systems. Both N- and C-terminals were flexible, as is commonly observed for globular proteins. Residues 13 to 23, which constitute a loop directly involved in ligand binding, showed ligand-induced fluctuations. These residues showed consistent interactions with act-D during its partial exit from the binding pocket of PknB, which resulted in slightly higher RMSF values compared to those observed for act-X_2_.

**Table 2:** Mean ± SD for RMSD_Protein_, RMSD_Ligand_, Center of mass distance between Protein-Ligand (CoM_Protein−Ligand_) and radius of gyration g_(r)_ for protein kinase (PknB) in complex with act-D and act-X_2_ ligands. The mean values have been calculated over the last 225 ns of 250 ns production run.

**Protein kinase PknB in complex with:**	**RMSD**_**Protein**_ **(Å)**	**RMSD**_**Ligand**_ **(Å)**	**CoM**_*Protein*−*Ligand*_ **Distance (Å)**	**g**_**(r)**_ **(Å)**
Act-D	2.59 ± 0.31	1.77 ± 0.29	18.17 ± 2.79	19.68 ± 0.20
Act-X_2_	2.85 ± 0.62	1.87 ± 0.27	15.96 ± 0.87	19.53 ± 0.27

**Table 3:** MM/GBSA binding energy in kcal/mol for protein-ligand complexes (protein kinase PknB in complex with Act-D and Act-X2). (A) MM/GBSA binding energy in kcal/mol for protein-ligand complexes (protein kinase PknB in complex with Act-D and Act-X2) averaged over 200 frames taken at the interval of 1ns over the last 200 ns of 250 ns production run. (B) MM/GBSA binding energy in kcal/mol for proteinligand complexes (protein kinase PknB in complex with Act-D and Act-X2) averaged over 50 frames taken at the interval of 1ns from 50 ns production run for 10 replicates. The different starting conformation for each simulation was taken from 250 ns long trajectory with an interval of 50 ns.

(A)					
**Protein kinase PknB in complex with:**	**ΔE** ^**V DW**^ **(van der Waal’s Energy)**	**ΔE** ^**elec**^ **(Coulombic Energy)**	**ΔG** ^**GB**^ **(Generalized-Born Polar Solvation Energy)**	**ΔG** ^**SASA**^ **(Non-Polar Solvation Energy)**	**ΔG** ^**MM/GBSA**^ **(Protein-Ligand Binding Energy)**
Act-D	−35.30 ± 10.98	−11.18 ± 8.76	30.94 ± 12.00	−3.50 ± 1.26	−19.04 ± 7.29
Act-X_2_	−57.12 ± 6.62	−6.26 ± 7.61	44.10 ± 7.43	−6.81 ± 0.76	−26.10 ± 4.93

(B)						
**Protein kinase PknB in complex with:**		**ΔE** ^**V DW**^ **(van der Waal’s Energy)**	**ΔE** ^**elec**^ **(Coulombic Energy)**	**ΔG** ^**GB**^ **(Generalized-Born Polar Solvation Energy)**	**ΔG** ^**SASA**^ **(Non-Polar Solvation Energy)**	**ΔG** ^**MM/GBSA**^ **(Protein-Ligand Binding Energy)**
Act-D	Replicate 1	−22.73	−16.65	27.62	−2.83	−14.58 ± 10.80
	Replicate 2	−52.93	−31.73	53.30	−84.66	−37.48 ± 10.23
	Replicate 3	−37.09	−14.65	36.69	−4.72	−19.77 ± 6.58
	Replicate 4	−19.62	−13.70	24.26	−2.37	−11.43 ± 8.25
	Replicate 5	−11.53	−3.32	9.73	−1.43	−6.55 ± 9.53
Act-X_2_	Replicate 1	−42.88	−14.99	37.60	−5.27	−25.54 ± 9.54
	Replicate 2	−42.56	−38.06	54.77	−5.47	−31.33 ± 11.07
	Replicate 3	−33.94	−22.96	41.74	−3.90	−19.06 ± 3.63
	Replicate 4	−23.40	−26.42	33.44	−3.05	−19.42 ± 10.32
	Replicate 5	−62.65	−38.74	71.08	−7.79	−38.10 ± 14.87

Residues 157 to 180 form a loop when PknB is in complex with act-X_2,_ and this is comparatively more flexible than the complex with actinomycin D. This might be due to the reduction in contact time caused by the partial exit of act-D from the binding pocket of PknB, as mentioned earlier. Another significant fluctuation can be observed for residues 78 to 89, corresponding to another loop in the proximity of the N-terminal. Overall, the majority of protein regions remained stable during the simulation time, with fluctuations of approximately 2 Å.

Figures 5A–5B displays the number of hydrogen bonds formed during the simulation between PknB and ligands, actinomycins X_2_ (red line) and D (black line). Act-X_2_ formed consistent H-bonds with PknB throughout the simulation. In contrast, act-D only formed H-bonds during ∼80 ns to 175 ns of the simulation, when it partially exited the binding pocket of PknB. In fact, act-D seldom forms H-bonds with PknB. After 175 ns of simulation, the ligand formed a greater number of H-bonds with the protein, which may indicate that act-D and PknB have a slower binding rate than act-X_2_ to reach the same efficacy; however, a detailed kinetic study would be needed to confirm this.

Figure 5C displays the radial distribution functions of ligands actinomycins X_2_ (red line) and D (black line), around the PknB protein. The peak maxima for the radial distribution of act-X_2_ lies close to 12.39 Å (higher intensities representing increased complex stability). A slight shoulder peak around 16.06 Å was observed, indicating a different distance for the protein-ligand complex. The peak maxima for the act-D-protein RDFs lie higher, close to 18.96 Å. However, an indistinguishable shoulder at a lower distance was also visible. Both RDFs show broad peaks, indicating that the ligands are flexible within the protein binding pocket.

Figure 5D shows the partial density of PknB in complex with actinomycins X_2_ (solid red line for protein and dashed line for ligand) and D (solid black line for protein and dashed line for ligand). No significant changes in the partial protein density were observed for either protein system. However, substantial changes in the distance between the peak maxima were noted for the partial density of ligands and their corresponding protein changes, indicating that the ligands may bind different regions of the binding site.

Figure 5E shows the molecular mechanics/Generalized Born surface area (MM/GBSA) ligand-protein binding energies. The energy contributions are given in Table 3A. The binding performance was better between Act-X_2_ and PknB (ΔG^MM/GBSA^ = −26.10 ± 4.93 kcal/mol) compared with Act-D and PknB. Act-D initially showed a higher ΔG, however, after 175 ns the binding energy was equivalent to that of act-X_2._ The mean ΔG^MM/GBSA^ value was −19.04 ± 7.29 kcal/mol. This shows that, although act-D has the same potential to bind PknB as act-X_2_, its binding reaction rate is slower; however, further kinetics studies would be required to confirm this.

Van der Waals forces are essential for favorable ligand-protein binding, while polar solvation free energy is highly unfavorable. Coulombic energy and non-polar solvation free energy are also favorable for ligand-protein binding. To assess ligand binding, 10 different replicates of act-D and act-X_2_ were tested to obtain 50 ns long trajectories for each simulation. These results complement our previous observations for both ligands (Table 3B).

Our *in silico* data supported the anti-TB activity we observed *in vitro*. The *in silico* data demonstrated that both actinomycins X_2_ and D had potential anti-TB activity against PknB. Act-X_2_ exhibited stronger binding affinity with faster kinetics, while act-D took longer to stabilize in the protein binding site with a slower reaction rate; although, further kinetics studies would be needed to confirm this.

## Discussion

MDR-TB is a highly contagious disease caused by MDR-Mtb. Due to the increasing prevalence of MDR in Mtb, treatment of MDR-TB is one of the most challenging issues affecting healthcare. Discovery of novel anti-TB drug candidates and targets is therefore essential to overcome these challenges. We therefore aimed to investigate the anti-TB efficacy of actinomycins, X_2_ and D (obtained from the novel *S. smyrnaeus* strain UKAQ_23) *in vitro,* and to use *in silico* studies to further support these findings. The Mtb protein kinase B (PknB) has therapeutic potential—particularly as it plays a role in cell growth regulation and cell wall synthesis, and it induces morphological changes during bacterial cell division (Wehenkel et al., 2008; Khan, Kaur & Nandicoori, 2018). In addition, PknB exhibited the best binding affinity with both actinomycins X_2_ and D. Therefore, PknB complexes with actinomycins X_2_ and D were studied using molecular dynamics (MD) simulations to investigate their structure and dynamics, and to assess their potential for inhibiting PknB function. The simulation trajectories were further subjected to molecular mechanics/Generalized Born surface area (MM/GBSA) binding energy evaluations to find ligand-protein binding interactions and associated thermodynamics. Earlier, several studies exploited MD simulation for the development of potential antitubercular drug targets/molecules (Almeleebia et al., 2021; Burley et al., 2021). The *in vitro* anti-TB activity of act-X_2_ and act-D was determined using the MABA method (the most widely adaptable method). We used three *Mycobacterium* strains: Mtb H37Ra, BCG, and Mtb H37Rv. Our results are consistent with previous studies of other strains that produce act-X_2_ and act-D (*i.e., Streptomyces* sp. IMB094, *Streptomyces* sp. MS449, *S.nasri* YG62, *S.elizabethii*. II, *S.padanus* JAU4234, *S.griseoruber*, *S.flavogriseus* NJ-4, *S.heliomycini*, *Streptomyces* strain M7, *S.hydrogenans* IB310, *Streptomyces* MITKK-103, and *Streptomyces* sp. HUST012) (Kurosawa et al., 2006; Bird & Latif, 2007; El-Naggar, El-Assar & Abdul-Gawad, 2009; Praveen & Tripathi, 2009; Chen et al., 2012; Xiong et al., 2012; Khieu et al., 2015; Wei et al., 2017; Kulkarni et al., 2017; Wang et al., 2017a; Wang et al., 2017b; Sharma & Manhas, 2019). In addition, several previous reports demonstrated that act-X_2_ and act-D had significant anti-TB activity (Praveen & Tripathi, 2009; Chen et al., 2012). Chen et al. (2012) reported the anti-TB activity of act-X_2_ and act-D produced by *Streptomyces* sp. MS449 against BCG and Mtb H37Rv. They reported MIC values of 0.5 µg/mL for act-X_2_ and 0.5 µg/mL for act-D against BCG; and 1.0 µg/mL for act-X_2_ and 8.0 µg/mL for act-D against Mtb H37Rv (Chen et al., 2012). These findings are consistent with our results, demonstrating the substantial anti-TB activity of isolated act-X_2_ and act-D against BCG and Mtb H37Rv. Another report demonstrated a MIC value of 0.78 µg/mL for the anti-TB activity of Py2 (act-D) against Mtb H37Rv, which is also consistent with our findings and further supports the anti-TB activity of act-X_2_ and act-D against Mtb H37Rv (Praveen & Tripathi, 2009). Although these earlier studies demonstrated actinomycins X_2_ and D showed anti-TB potential, they did not perform any *in silico* anti-TB investigations. Our study demonstrates that the act-X_2_ and act-D, produced by the novel *S. smyrnaeus* strain UKAQ_23, exhibit substantial anti-TB activity against the *Mycobacterium* strains tested. In addition, we have demonstrated that isolated act-X_2_ and act-D had comparable anti-TB efficiency against the Mtb H37Ra and BCG strains; although, act-D exhibited significantly higher anti-TB activity than act-X_2_ against the Mtb H37Rv strain.

## Conclusions

In conclusion, we suggest that both actinomycins X_2_ and D, (newly sourced from the novel *S. smyrnaeus* strain UKAQ_23), are highly potent anti-TB drug candidates. Our results indicate that act-X_2_ is more potent than act-D, and thus has more potential to be used as a new anti-TB drug candidate—by antagonistically interacting with the kinase PknB target protein.

##  Supplemental Information

10.7717/peerj.14502/supp-1 Supplemental Information 1 Supplemental Figures and Tables

10.7717/peerj.14502/supp-2 Supplemental Information 2 Raw data

10.7717/peerj.14502/supp-3 Supplemental Information 3 Supplimentary_file-3

## Additional Information and Declarations

### Data Availability

The following information was supplied regarding data availability: The raw data is available in the Supplemental Files.

## References

[ref-1] Abraham MJ, Murtola T, Schulz R, Páll S, Smith JC, Hess B, Lindah E (2015). Gromacs: high performance molecular simulations through multi-level parallelism from laptops to supercomputers. SoftwareX.

[ref-2] Ali M, Shaw DR, Zhang L, Haroon MF, Narita Y, Emwas AH, Saikaly PE, Okabe S (2018). Aggregation ability of three phylogenetically distant anammox bacterial species. *Water Research*.

[ref-3] Almeleebia TM, AlShahrani M, Alshahrani MY, Ahmad I, Alkahtani AM, Alam MJ, Kausar MA, Saeed A, Saeed M, Iram S (2021). Identification of new mycobacterium tuberculosis proteasome inhibitors using a knowledge-based computational screening approach. Molecules.

[ref-4] Amin AG, Angala SK, Chatterjee D, Crick DC (2008). Rapid screening of inhibitors of mycobacterium tuberculosis growth using tetrazolium salts. *Methods in Molecular Biology*.

[ref-5] Anandakrishnan R, Aguilar B, Onufriev AV (2012). H++ 3.0: automating pK prediction and the preparation of biomolecular structures for atomistic molecular modeling and simulations. *Nucleic Acids Research*.

[ref-6] Azam F, Eid EEM, Almutairi A (2021). Targeting SARS-CoV-2 main protease by teicoplanin: a mechanistic insight by docking, MM/GBSA and molecular dynamics simulation. *Journal of Molecular Structure*.

[ref-7] Baptista R, Bhowmick S, Shen J, Mur LAJ (2021). Molecular docking suggests the targets of anti-mycobacterial natural products. Molecules.

[ref-8] Bird CW, Latif M (2007). Antibiotics from the newly isolated Streptomyces elizabethii, II. Isolation and characterisation of the antibiotics. *Journal of Chemical Technology and Biotechnology*.

[ref-9] Burley KH, Cuthbert BJ, Basu P, Newcombe J, Irimpan EM, Quechol R, Foik IP, Mobley DL, Beste DJV, Goulding CW (2021). Structural and molecular dynamics of mycobacterium tuberculosis malic enzyme, a potential anti-TB drug target. ACS Infectious Diseases.

[ref-10] Chawla Y, Upadhyay S, Khan S, Nagarajan SN, Forti F, Nandicoori VK (2014). Protein kinase B (PknB) of Mycobacterium tuberculosis is essential for growth of the pathogen in vitro as well as for survival within the host. *Journal of Biological Chemistry*.

[ref-11] Chen C, Song F, Wang Q, Abdel-Mageed WM, Guo H, Fu C, Hou W, Dai H, Liu X, Yang N, Xie F, Yu K, Chen R, Zhang L (2012). A marine-derived Streptomyces sp. MS449 produces high yield of actinomycin X2 and actinomycin D with potent anti-Tuberculosis activity. *Applied Microbiology and Biotechnology*.

[ref-12] Chen Z, Ou P, Liu L, Jin X (2020). Anti-MRSA activity of actinomycin X2 and collismycin a produced by streptomyces globisporus WA5-2-37 from the intestinal tract of American Cockroach (Periplaneta americana). *Frontiers in Microbiology*.

[ref-13] Cho S, Lee HS, Franzblau S (2015). Microplate alamar blue assay (MABA) and Low oxygen recovery assay (LORA) for Mycobacterium tuberculosis. *Methods in Molecular Biology*.

[ref-14] Collins LA, Franzblau SG (1997). Microplate Alamar blue assay versus BACTEC 460 system for high- throughput screening of compounds against Mycobacterium tuberculosis and Mycobacterium avium. *Antimicrobial Agents and Chemotherapy*.

[ref-15] El-Naggar MY, El-Assar SA, Abdul-Gawad SM (2009). Solid-state fermentation for the production of meroparamycin by Streptomyces sp. strain MAR01. *Journal of Microbiology and Biotechnology*.

[ref-16] Franzblau SG, Witzig RS, Mclaughlin JC, Torres P, Madico G, Hernandez A, Degnan MT, Cook MB, Quenzer VK, Ferguson RM, Gilman RH (1998). Rapid, low-technology MIC determination with clinical Mycobacterium tuberculosis isolates by using the microplate Alamar Blue assay. *Journal of Clinical Microbiology*.

[ref-17] Huang J, Rauscher S, Nawrocki G, Ran T, Feig M, De Groot BL, Grubmüller H, MacKerell AD (2016). CHARMM36m: An improved force field for folded and intrinsically disordered proteins. *Nature Methods*.

[ref-18] Hussain MS, Azam F, Eldarrat HA, Alkskas I, Mayoof JA, Dammona JM, Ismail H, Ali M, Arif M, Haque A (2020). Anti-inflammatory, analgesic and molecular docking studies of Lanostanoic acid 3-O- *α*-D-glycopyranoside isolated from Helichrysum stoechas. *Arabian Journal of Chemistry*.

[ref-19] Jamal Q (2020). Antileishmanial, cytotoxic and genotoxic effects of actinomycin D, Z3, Z5 and hydrazine derivatives of isosteviol. http://prr.hec.gov.pk/jspui/handle/123456789/12101.

[ref-20] Jiang H, Liu M, Zhang Y, Yin J, Li Z, Zhu C, Li Q, Luo X, Ji T, Zhang J, Yang Y, Wang X, Luo Y, Tao L, Zhang F, Liu X, Li W, Guo X (2021). Changes in incidence and epidemiological characteristics of pulmonary tuberculosis in Mainland China. JAMA Network Open.

[ref-21] Jo S, Kim T, Iyer VG, Im W (2008). CHARMM-GUI: a web-based graphical user interface for CHARMM. *Journal of Computational Chemistry*.

[ref-22] Khan MZ, Kaur P, Nandicoori VK (2018). Targeting the messengers: serine/threonine protein kinases as potential targets for antimycobacterial drug development. *IUBMB Life*.

[ref-23] Khieu TN, Liu MJ, Nimaich S, Quach NT, Chu-Ky S, Phi QT, Vu TT, Nguyen TD, Xiong Z, Prabhu DM, Li WJ (2015). Characterization and evaluation of antimicrobial and cytotoxic effects of Streptomyces sp. HUST012 isolated from medicinal plant Dracaena cochinchinensis Lour. *Frontiers in Microbiology*.

[ref-24] Kulkarni M, Gorthi S, Banerjee G, Chattopadhyay P (2017). Production, characterization and optimization of actinomycin D from Streptomyces hydrogenans IB310, an antagonistic bacterium against phytopathogens. *Biocatalysis and Agricultural Biotechnology*.

[ref-25] Kurosawa K, Bui VP, Van Essendelft JL, Willis LB, Lessard PA, Ghiviriga I, Sambandan TG, Rha CK, Sinskey AJ (2006). Characterization of Streptomyces MITKK-103, a newly isolated actinomycin X2-producer. *Applied Microbiology and Biotechnology*.

[ref-26] Lee J, Cheng X, Swails JM, Yeom MS, Eastman PK, Lemkul JA, Wei S, Buckner J, Jeong JC, Qi Y, Jo S, Pande VS, Case DA, Brooks CL, MacKerell AD, Klauda JB, Im W (2016). CHARMM-GUI input generator for NAMD, GROMACS, AMBER, OpenMM, and CHARMM/OpenMM simulations using the CHARMM36 additive force field. *Journal of Chemical Theory and Computation*.

[ref-27] Mendoza-Aguilar M, Almaguer-Villagrán L, Jiménez-Arellanes A, Arce-Paredes P, Cid-Gutiérrez JL, Rojas-Espinosa O (2012). The use of the microplate alamar blue assay (MABA) to assess the susceptibility of Mycobacterium lepraemurium to anti-leprosy and other drugs. *Journal of Infection and Chemotherapy*.

[ref-28] Miller BR, McGee TD, Swails JM, Homeyer N, Gohlke H, Roitberg AE (2012). MMPBSA.py: an efficient program for end-state free energy calculations. *Journal of Chemical Theory and Computation*.

[ref-29] Müller Al (2016). TB online. https://www.tbonline.info/posts/author/6/.

[ref-30] Praveen V, Tripathi CKM (2009). Studies on the production of actinomycin-D by Streptomyces griseoruber - a novel source. *Letters in Applied Microbiology*.

[ref-31] Qureshi KA, Bholay AD, Rai PK, Mohammed HA, Khan RA, Azam F, Jaremko M, Emwas AH, Stefanowicz P, Waliczek M, Kijewska M, Ragab EA, Rehan M, Elhassan GO, Anwar MJ, Prajapati DK (2021a). Isolation, characterization, anti-MRSA evaluation, and in-silico multi-target anti-microbial validations of actinomycin X2 and actinomycin D produced by novel Streptomyces smyrnaeus UKAQ_23. Scientific Reports.

[ref-32] Qureshi KA, Nasr Al I, Koko WS, Khan TA, Fatmi MQ, Imtiaz M, Khan RA, Mohammed HA, Jaremko M, Emwas AH, Azam F, Bholay AD, Elhassan GO, Prajapati DK (2021b). In vitro and in silico approaches for the antileishmanial activity evaluations of actinomycins isolated from novel Streptomyces smyrnaeus strain UKAQ_23. *Antibiotics*.

[ref-33] Qureshi KA, Imtiaz M, Parvez A, Rai PK, Jaremko M, Emwas AH, Bholay AD, Fatmi MQ (2022). In vitro and in silico approaches for the evaluation of antimicrobial activity, time-kill kinetics, and anti-biofilm potential of Thymoquinone (2-Methyl-5-propan-2-ylcyclohexa-2, 5-diene-1,4-dione) against selected human pathogens. Antibiotics.

[ref-34] Sanasam S, Ningthoujam SD (2010). Screening of local actinomycete isolates in Manipur for anticandidal activity. *Asian Journal of Biotechnology*.

[ref-35] Sharma M, Manhas RK (2019). Purification and characterization of actinomycins from Streptomyces strain M7 active against methicillin resistant Staphylococcus aureus and vancomycin resistant Enterococcus. *BMC Microbiology*.

[ref-36] Shushni MAM, Azam F, Lindequist U (2013). Oxasetin from Lophiostoma sp. of the Baltic Sea: identification, in silico binding mode prediction and antibacterial evaluation against fish pathogenic bacteria. *Natural Product Communications*.

[ref-37] Singh LS, Baruah I, Bora TC (2006). Actinomycetes of Loktak habitat: isolation and screening for antimicrobial activities. *Biotechnology*.

[ref-38] Tanaka Y, Omura S (1993). Agroactive compounds of microbial origin. *Annual Review of Microbiology*.

[ref-39] Timo GO,  dos Reis RSSV, de Melo AF, Costa TVL, Magalhães de OP, Homem-de Mello M (2019). Predictive power of in silico approach to evaluate chemicals against m. tuberculosis: a systematic review. Pharmaceuticals.

[ref-40] Trott O, Olson AJ (2009). AutoDock Vina: improving the speed and accuracy of docking with a new scoring function, efficient optimization, and multithreading. Journal of Computational Chemistry.

[ref-41] Wang D, Wang C, Gui P, Liu H, Khalaf SMH, Elsayed EA, Wadaan MAM, Hozzein WN, Zhu W (2017a). Identification, bioactivity, and productivity of actinomycins from the marine-derived Streptomyces heliomycini. *Frontiers in Microbiology*.

[ref-42] Wang Q, Zhang Y, Wang M, Tan Y, Hu X, He H, Xiao C, You X, Wang Y, Gan M (2017b). Neo-actinomycins A and B, natural actinomycins bearing the 5H-oxazolo[4,5-b]phenoxazine chromophore, from the marine-derived Streptomyces sp. IMB094. *Scientific Reports*.

[ref-43] Wehenkel A, Bellinzoni M, Graña M, Duran R, Villarino A, Fernandez P, Andre-Leroux G, England P, Takiff H, Cerveñansky C, Cole ST, Alzari PM (2008). Mycobacterial Ser/Thr protein kinases and phosphatases: physiological roles and therapeutic potential. *Biochimica et Biophysica Acta - Proteins and Proteomics*.

[ref-44] Wehenkel A, Fernandez P, Bellinzoni M, Catherinot V, Barilone N, Labesse G, Jackson M, Alzari PM (2006). The structure of PknB in complex with mitoxantrone, an ATP-competitive inhibitor, suggests a mode of protein kinase regulation in mycobacteria. *FEBS Letters*.

[ref-45] Wei Z, Xu C, Wang J, Lu F, Bie X, Lu Z (2017). Identification and characterization of Streptomyces flavogriseus NJ-4 as a novel producer of actinomycin D and holomycin. PeerJ.

[ref-46] Xiong ZQ, Zhang ZP, Li JH, Wei SJ, Tu GQ (2012). Characterization of Streptomyces padanus JAU4234, a producer of actinomycin X2, fungichromin, and a new polyene macrolide antibiotic. *Applied and Environmental Microbiology*.

[ref-47] Zhang Z, Gao P, Guan Y, Xiao CL, Hao XQ (2009). Isolation, purification, identification of structure and study of bioactivity of anti-TB active component 9005B. *Chinese Journal of Antibiotics*.

