# Supplementary material for: In vitro and in silico evaluations of actinomycin X2and actinomycin D as potent anti-tuberculosis agents
Source: PeerJ. 2023 Mar 8;11:e14502. doi: 10.7717/peerj.14502 (PMC10022501; doi:10.7717/peerj.14502)
Supplement: Supplemental Information 1 [file peerj-11-14502-s001.docx]

***In vitro* and *in silico* evaluations of actinomycin X_2_ and actinomycin D as potent anti-tuberculosis agents**

Kamal A. Qureshi^1^,*, Faizul Azam^2^, M. Qaiser Fatmi^3^, Mahrukh Imtiaz^3^, Dinesh K. Prajapati^4^, Pankaj K. Rai^4^, Mariusz Jaremko^5^, Abdul-Hamid Emwas^6^, Gamal O. Elhassan^1^

^1^Department of Pharmaceutics, Unaizah College of Pharmacy, Qassim University, Unaizah-51911, Al-Qassim, Saudi Arabia

^2^Department of Pharmaceutical Chemistry and Pharmacognosy, Unaizah College of Pharmacy, Qassim University, Unaizah-51911, Al-Qassim, Saudi Arabia

^3^Department of Biosciences, COMSATS University Islamabad, Islamabad, Pakistan

^4^Department of Biotechnology, Faculty of Biosciences, Invertis University, Bareilly-243123, Uttar Pradesh, India

^5^Smart-Health Initiative (SHI) and Red Sea Research Center (RSRC), Division of Biological and Environmental Sciences and Engineering (BESE), King Abdullah University of Science and Technology (KAUST), Thuwal-23955-6900, Jeddah, Saudi Arabia

^6^Core Labs, King Abdullah University of Science and Technology (KAUST), Thuwal 23955‑6900, Saudi Arabia.

Corresponding Author:

Dr. Kamal A. Qureshi

Email address: [ka.qurishe@qu.edu.sa](mailto:ka.qurishe@qu.edu.sa); Phone: +966599110591

https://orcid.org/0000-0002-3498-797X

**Supplementary Information**


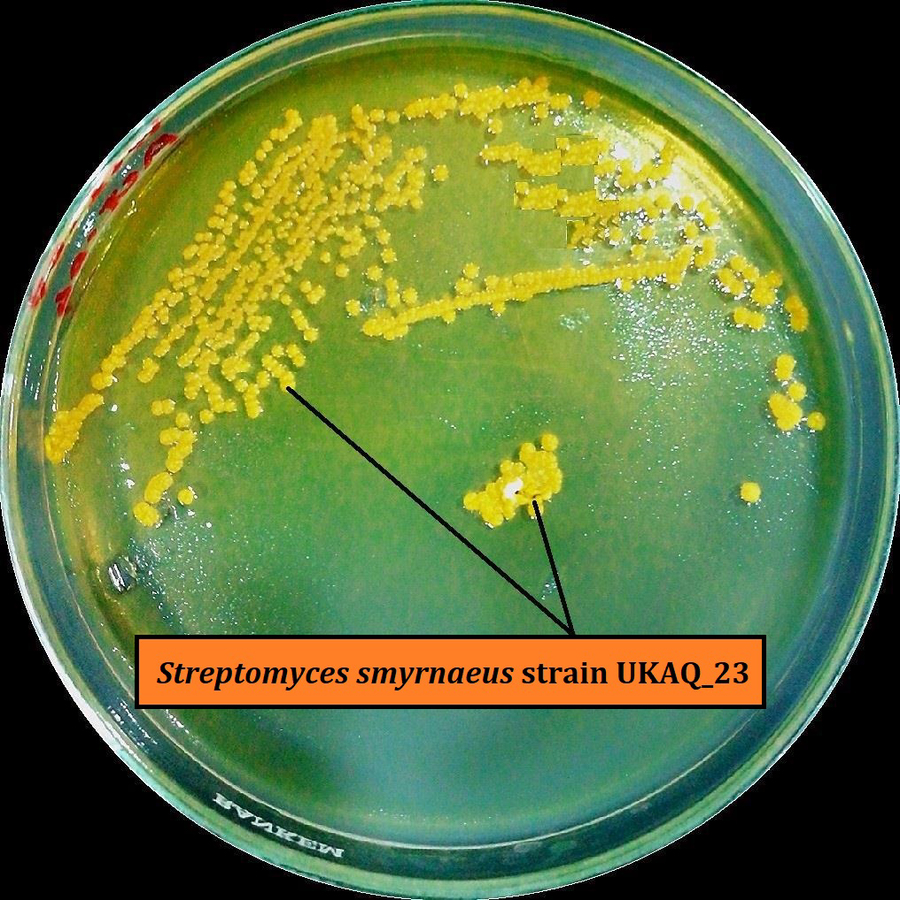


Figure S1. Growth of novel *S. smyrnaeus* strain UKAQ_23 on modified ISP-4 agar medium.


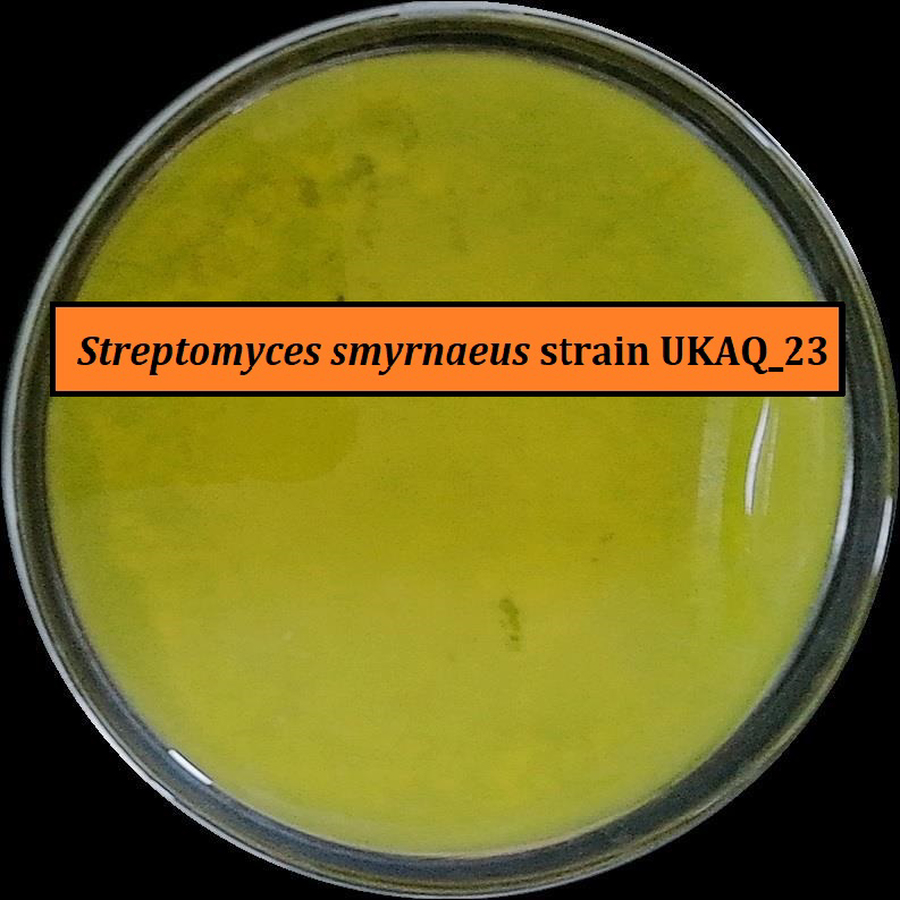


Figure S2. Production of actinomycins X_2_ and D by novel *S. smyrnaeus* strain UKAQ_23 in modified ISP-4 agar medium.


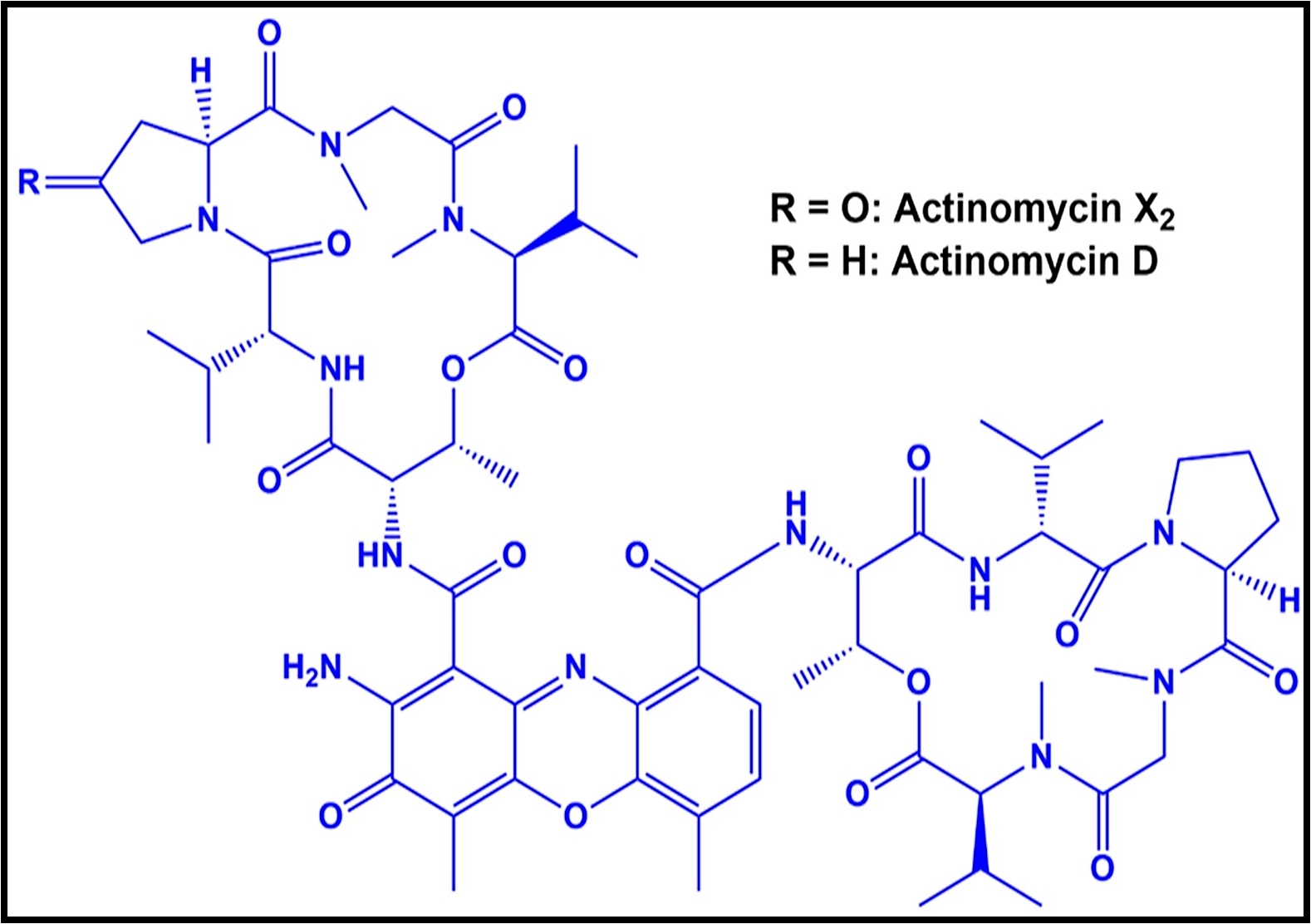


Figure S3. Structures of isolated actinomycins X_2_ and D.


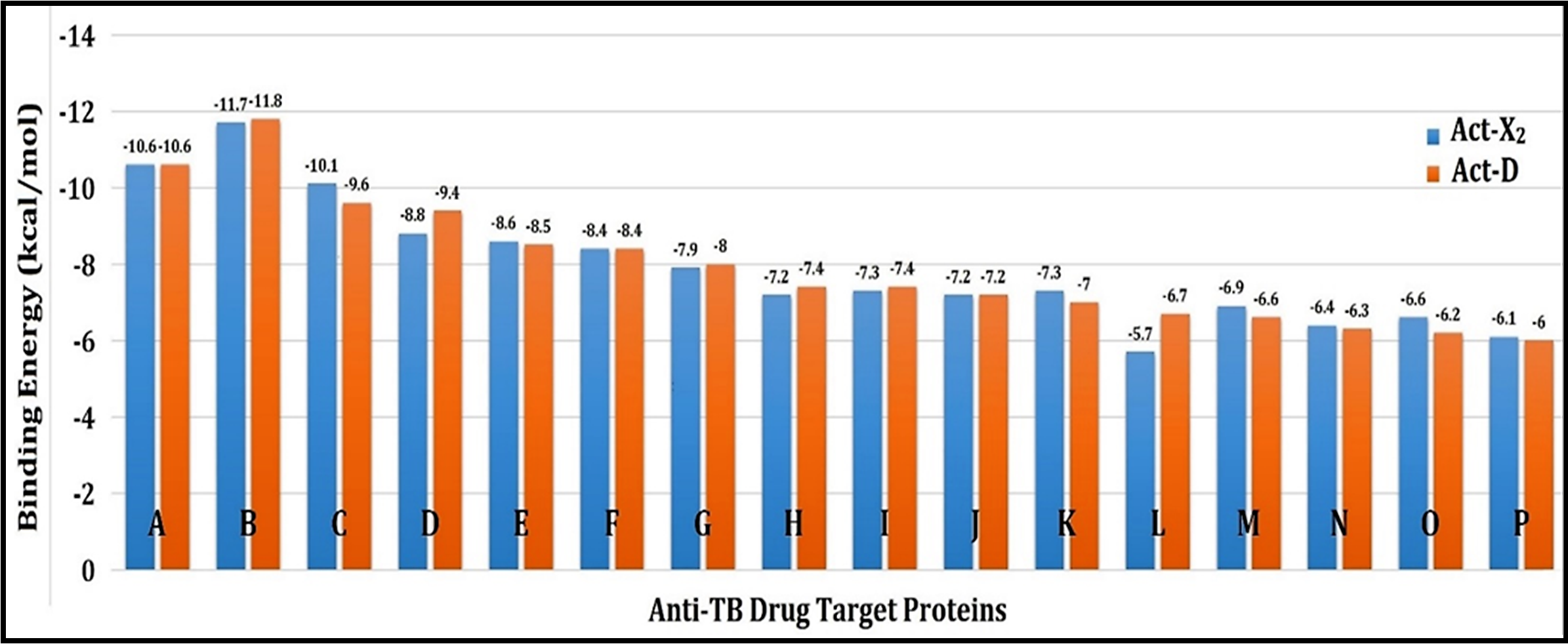


**Figure S4.** A bar plot of molecular docking-predicted binding energies in kcal/mol and several anti-TB drug targets.

**Note:** **A-**DNA; **B-**Protein kinase PknB; **C-**Polyketide synthase 13; **D-**Lumazine synthase; **E**-Pantothenate kinase **F-**DprE1; **G-**Protein tyrosine phosphatase PtpB; **H-**DNA GyrB ATPase domain; **I-**Enoyl reductase; **J-**DNA topoisomerase I; **K-**NAD^+^-dependent DNA ligase A; **L-**KasA; **M-**Mtb type II dehydroquinase; **N-**Diacylglycerol acyltransferase/mycolyltransferase Ag85C; **O-**Mtb shikimate kinase; **P-**Pantothenate synthetase.


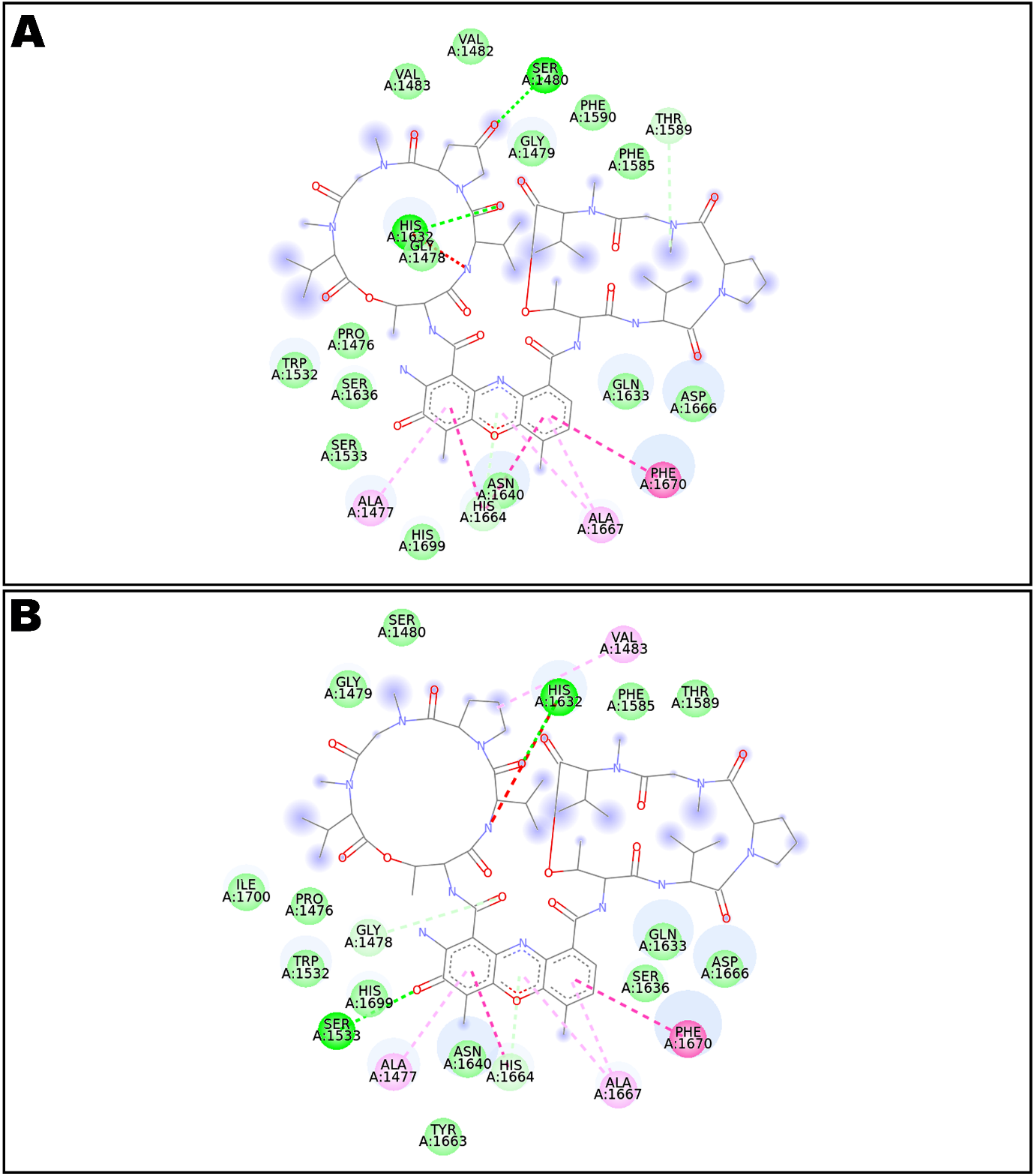


**Figure S5.** Non-bond interactions of act-X_2_ (A) and act-D (B) with polyketide synthase 13.


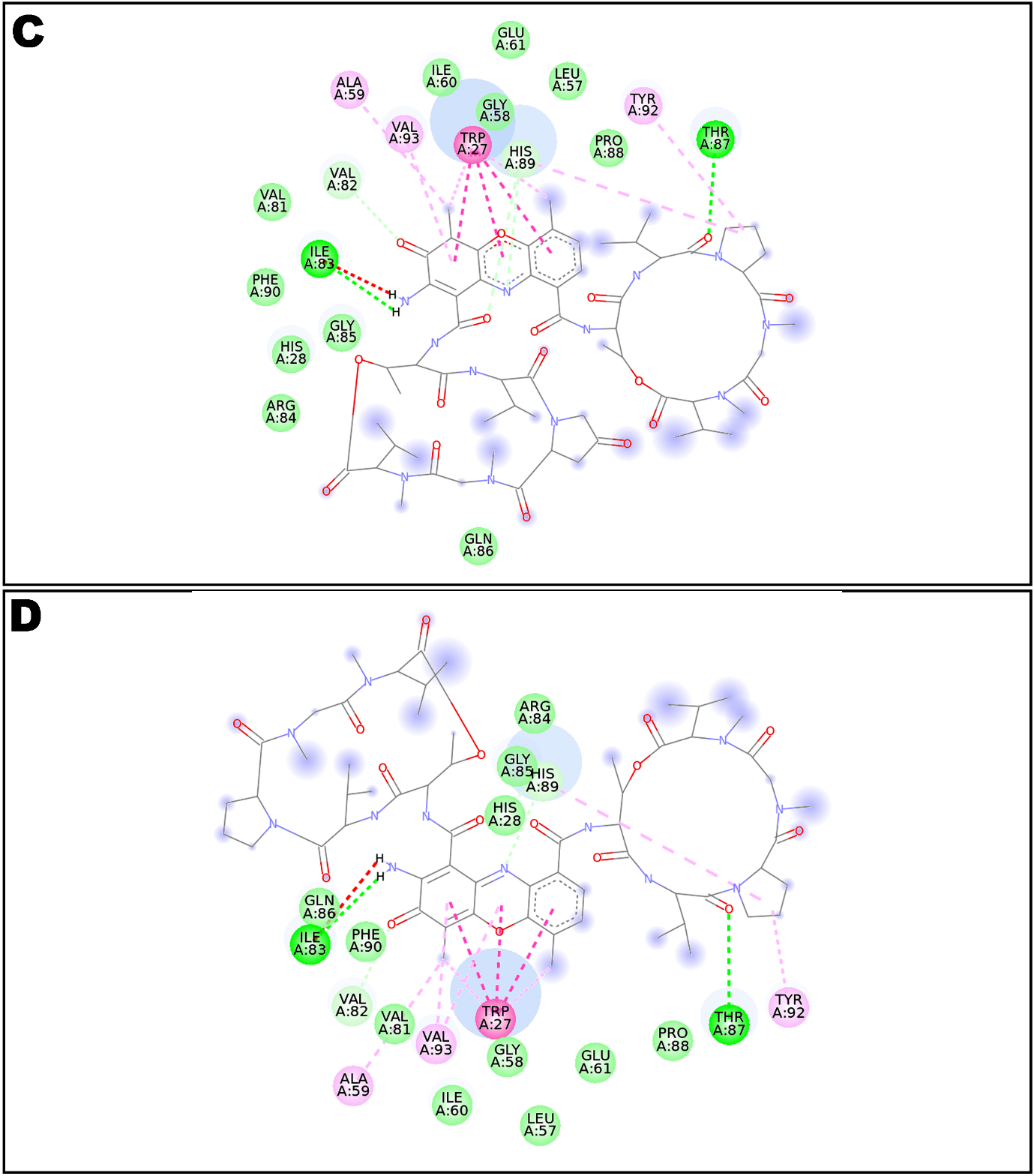


**Figure S6.** Intermolecular interactions of act-X_2_ (C) and act-D (D) with lumazine synthase.


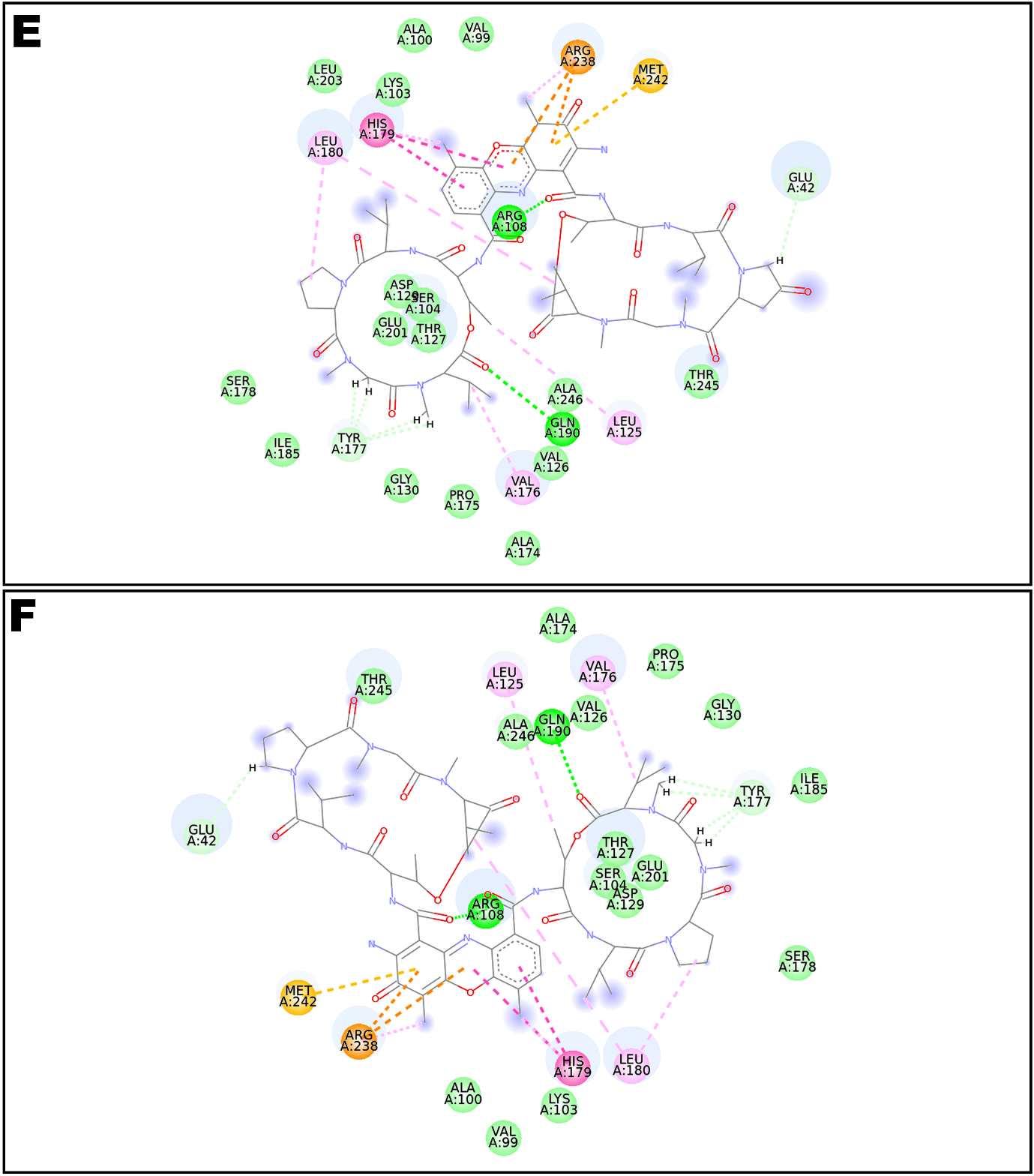


**Figure S7.** Docked act-X_2_ (E) and act-D (F) within inhibitor binding cavity of pantothenate kinase.


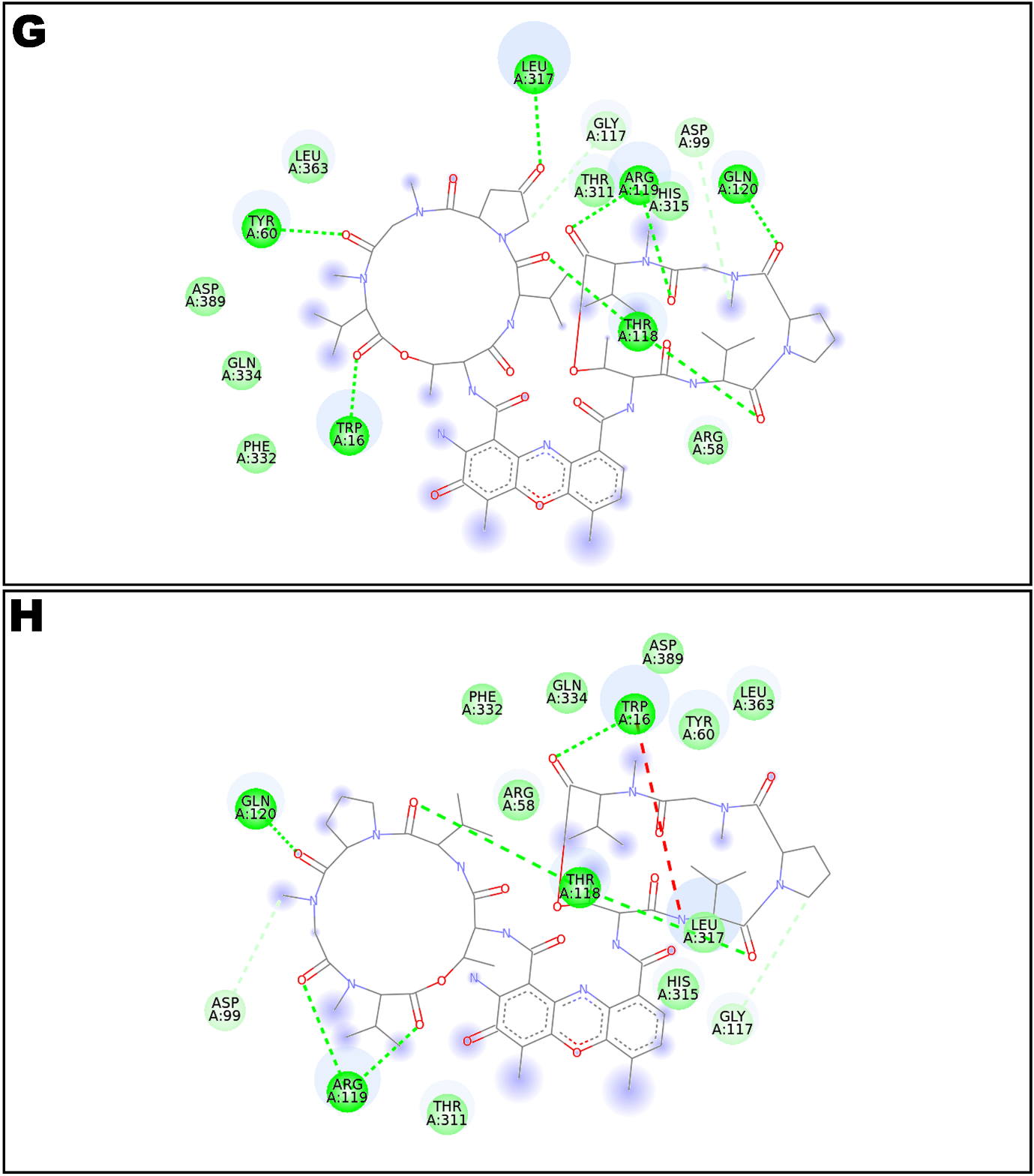


**Figure S8.** Non-bond interactions of act-X_2_ (G) and act-D (H) with decaprenylphosphoryl-β-D-ribose-2'-oxidase (DprE1).


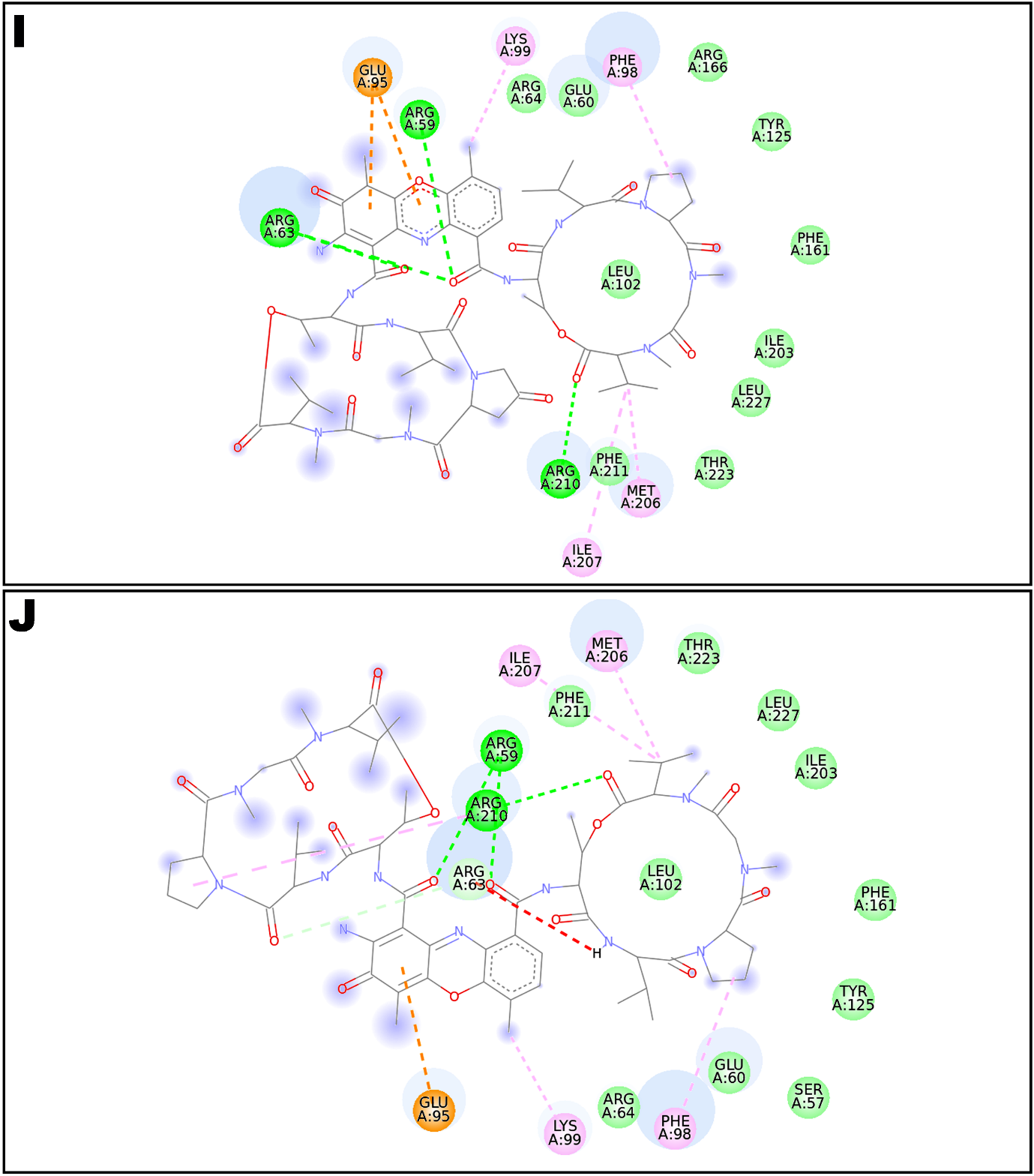


**Figure S9.** Non-bond interactions of act-X_2_ (I) and act-D (J) with protein tyrosine phosphatase PtpB.


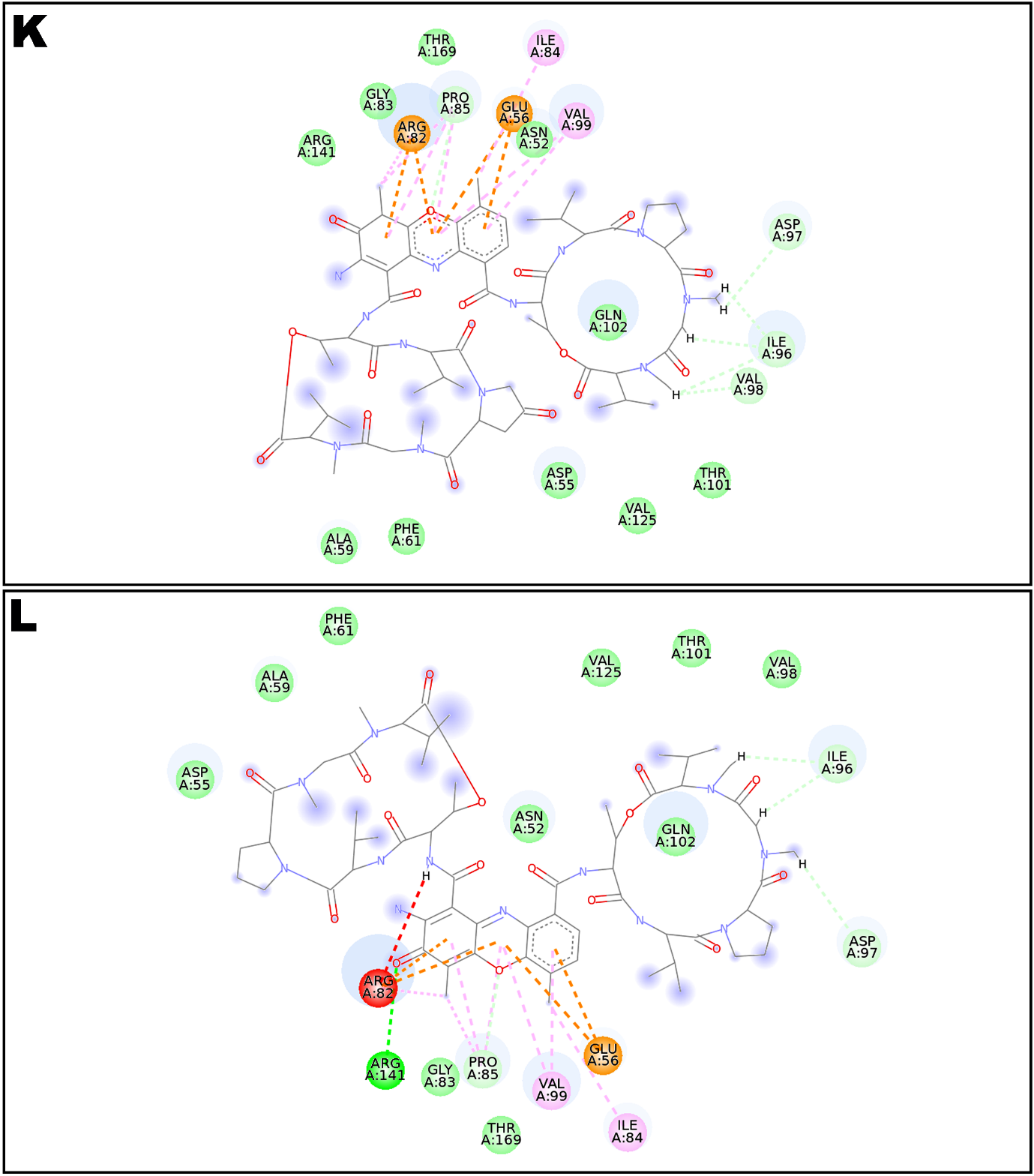


**Figure S10.** Non-bond interactions of act-X_2_ (K) and act-D (L) with DNA GyrB ATPase domain.


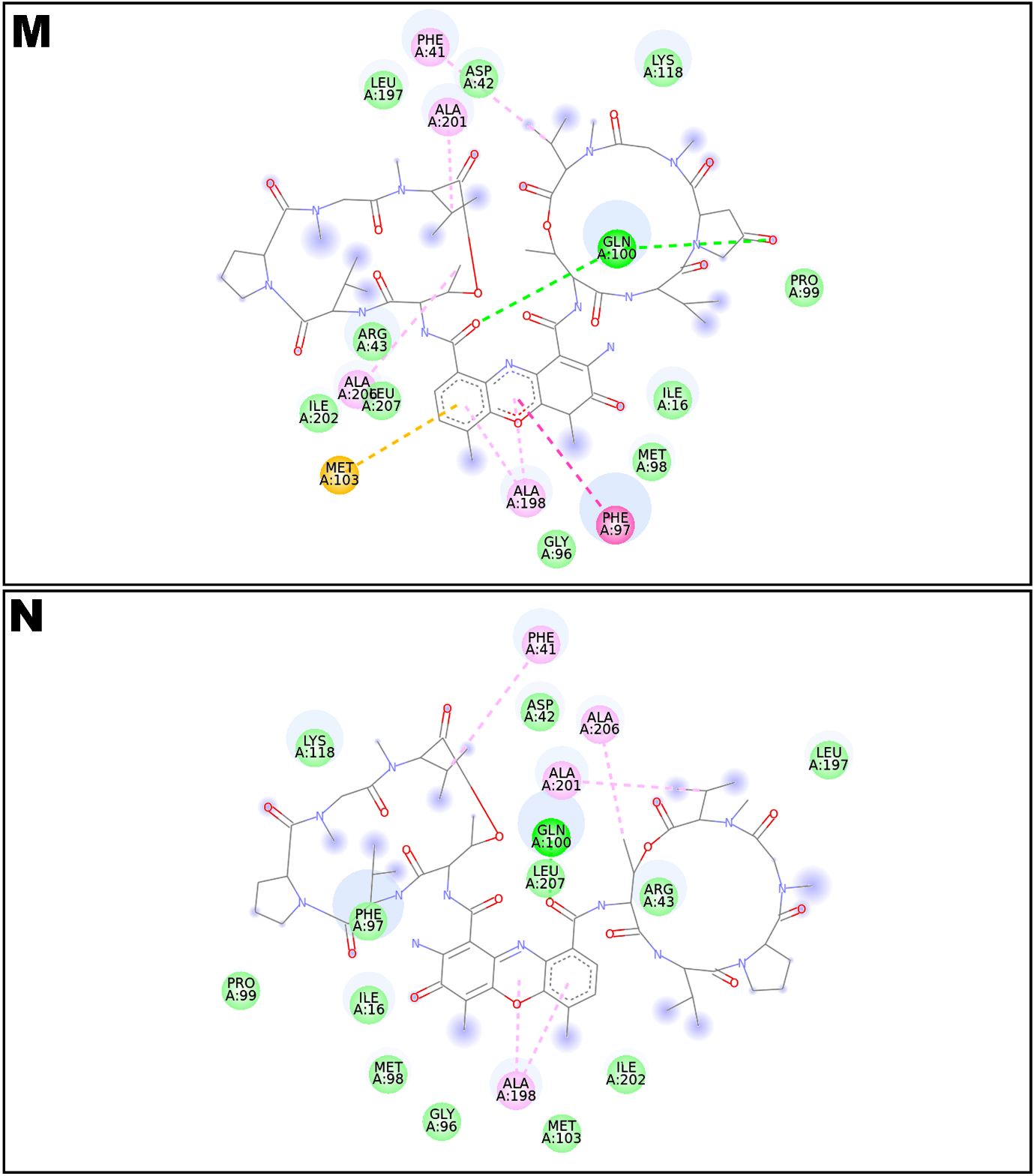


**Figure S11.** Non-bond interactions of act-X_2_ (M) and act-D (N) with enoyl reductase.


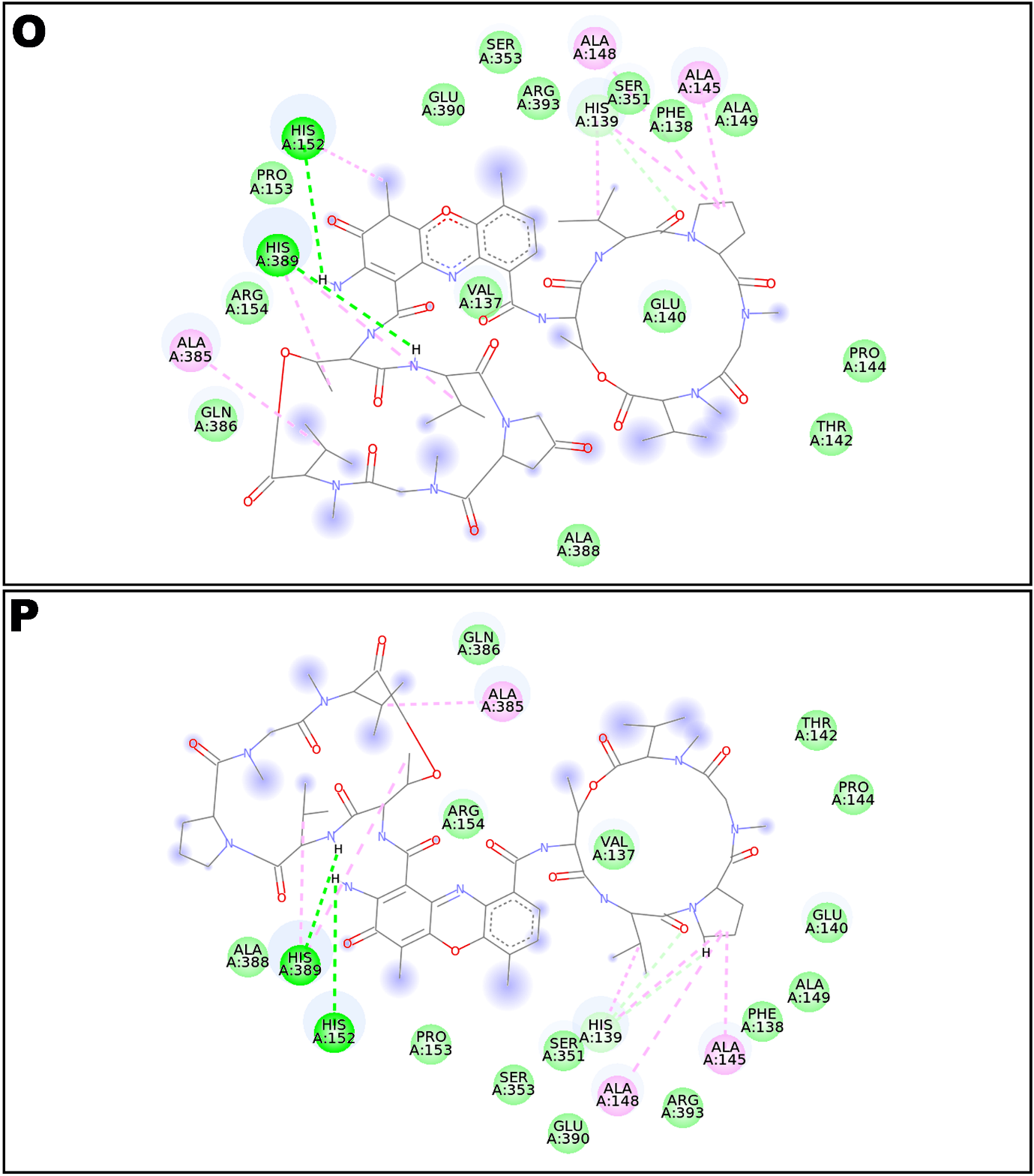


**Figure S12.** Non-bond interactions of act-X_2_ (O) and act-D (P) with DNA topoisomerase-I.


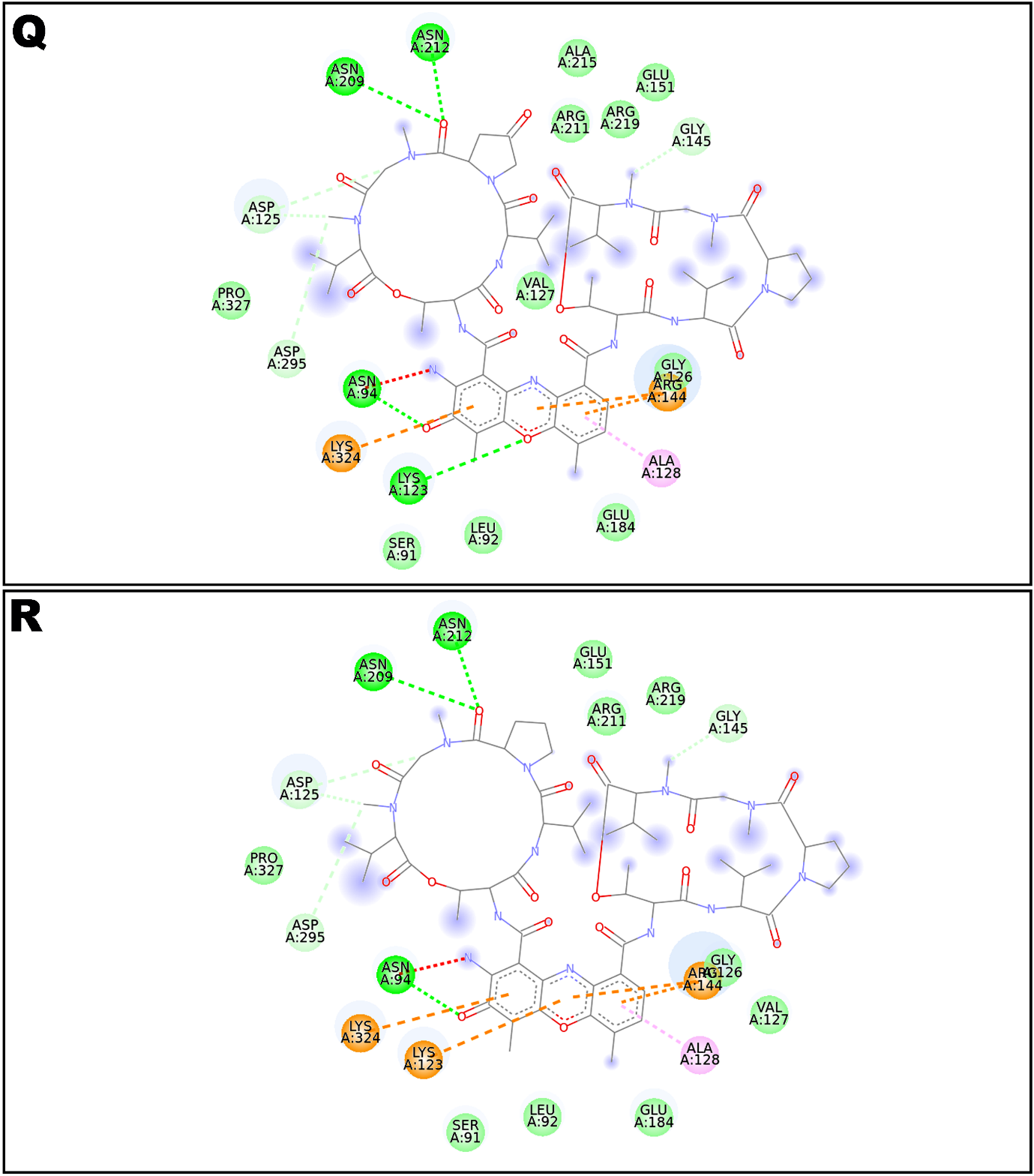


**Figure S13.** Non-bond interactions of act-X_2_ (Q) and act-D (R) with DNA ligase.


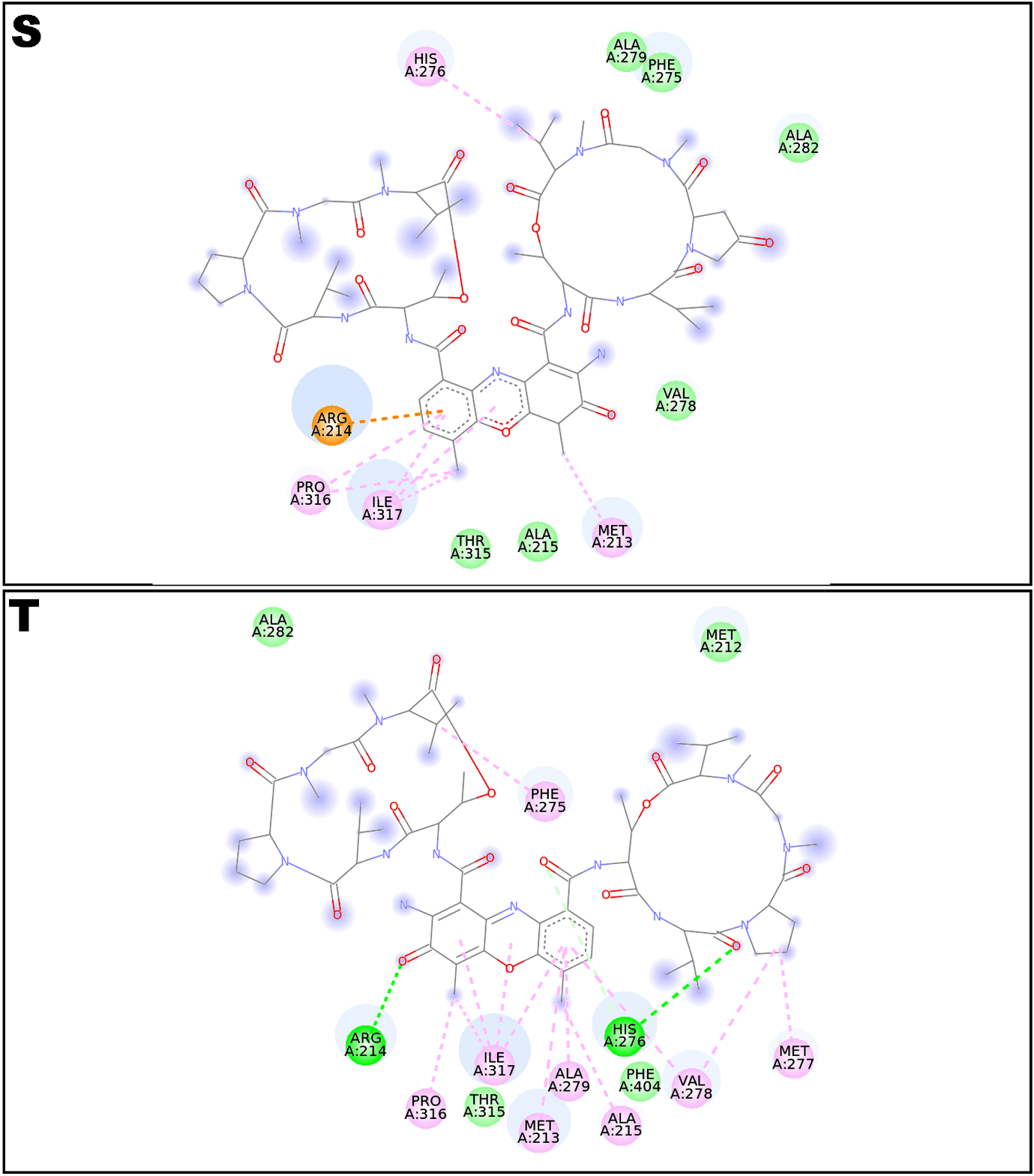


**Figure S14.** Non-bond interactions of act-X_2_ (S) and act-D (T) with β-ketoacyl synthase-A (KasA).


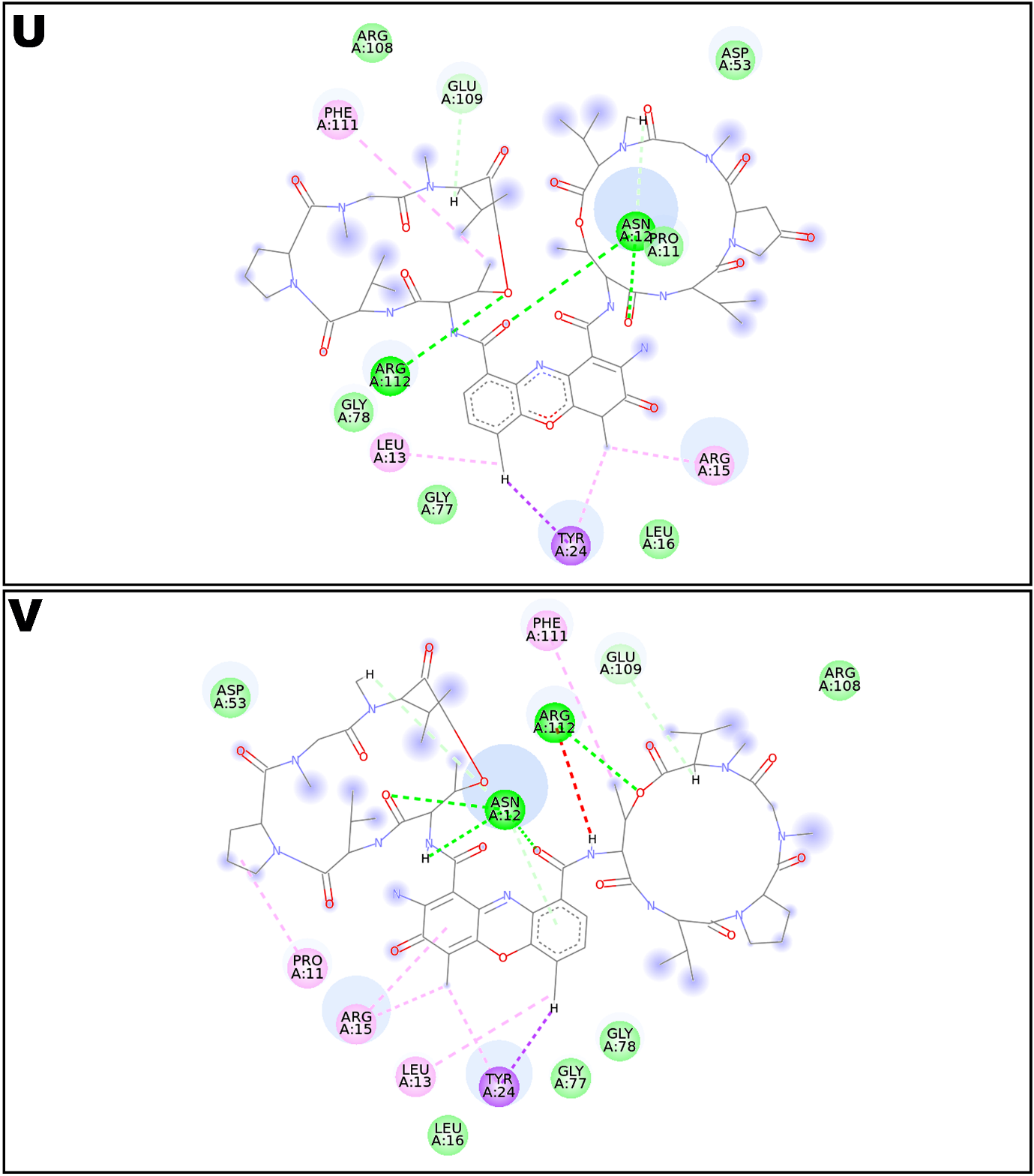


**Figure S15.** Non-bond interactions of act-X_2_ (U) and act-D (V) with mycobacterium type II dehydroquinase.


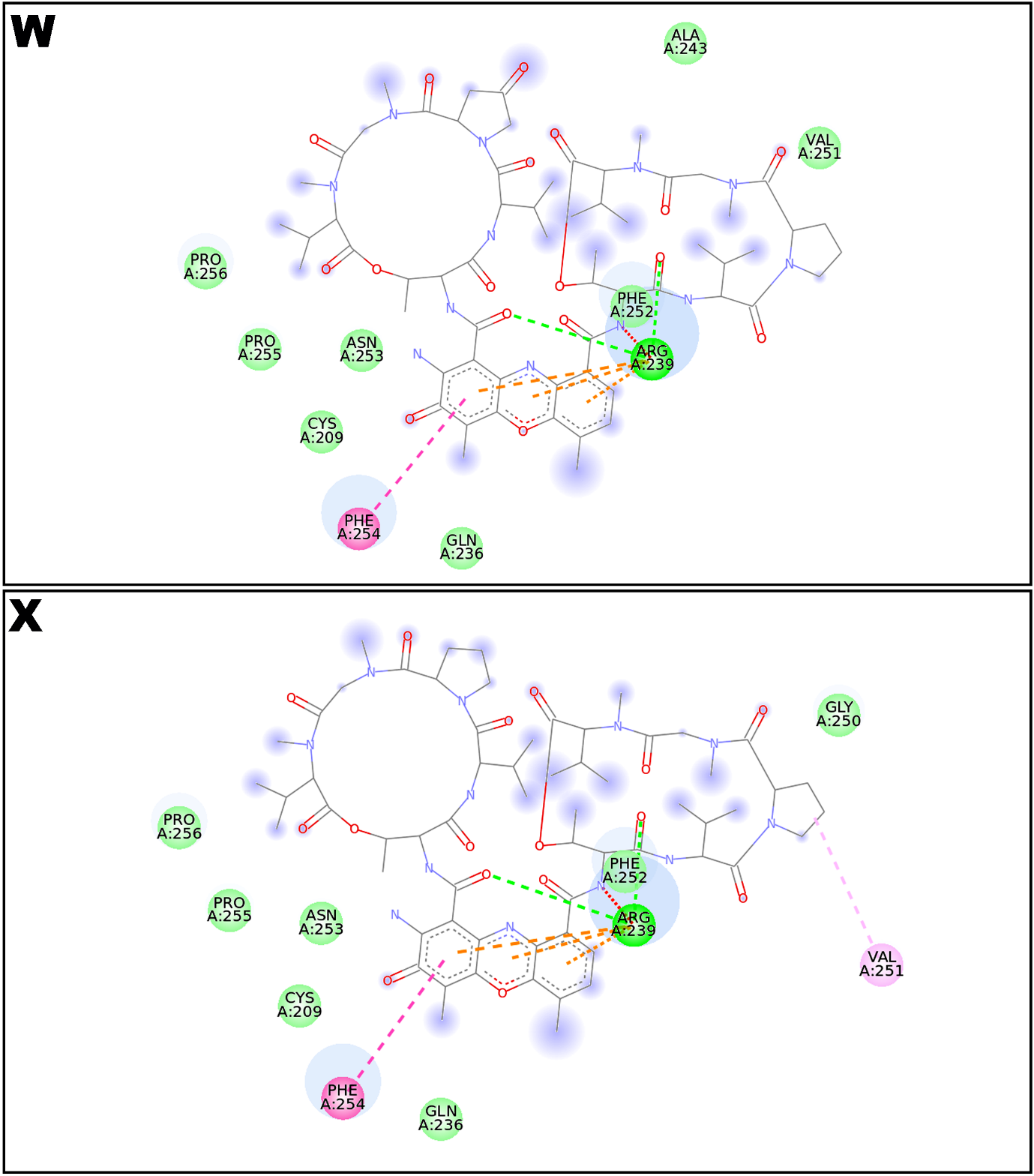


**Figure S16.** Non-bond interactions of act-X_2_ (W) and act-D (X) with Diacylglycerol acyltransferase/mycolyltransferase Ag85C.


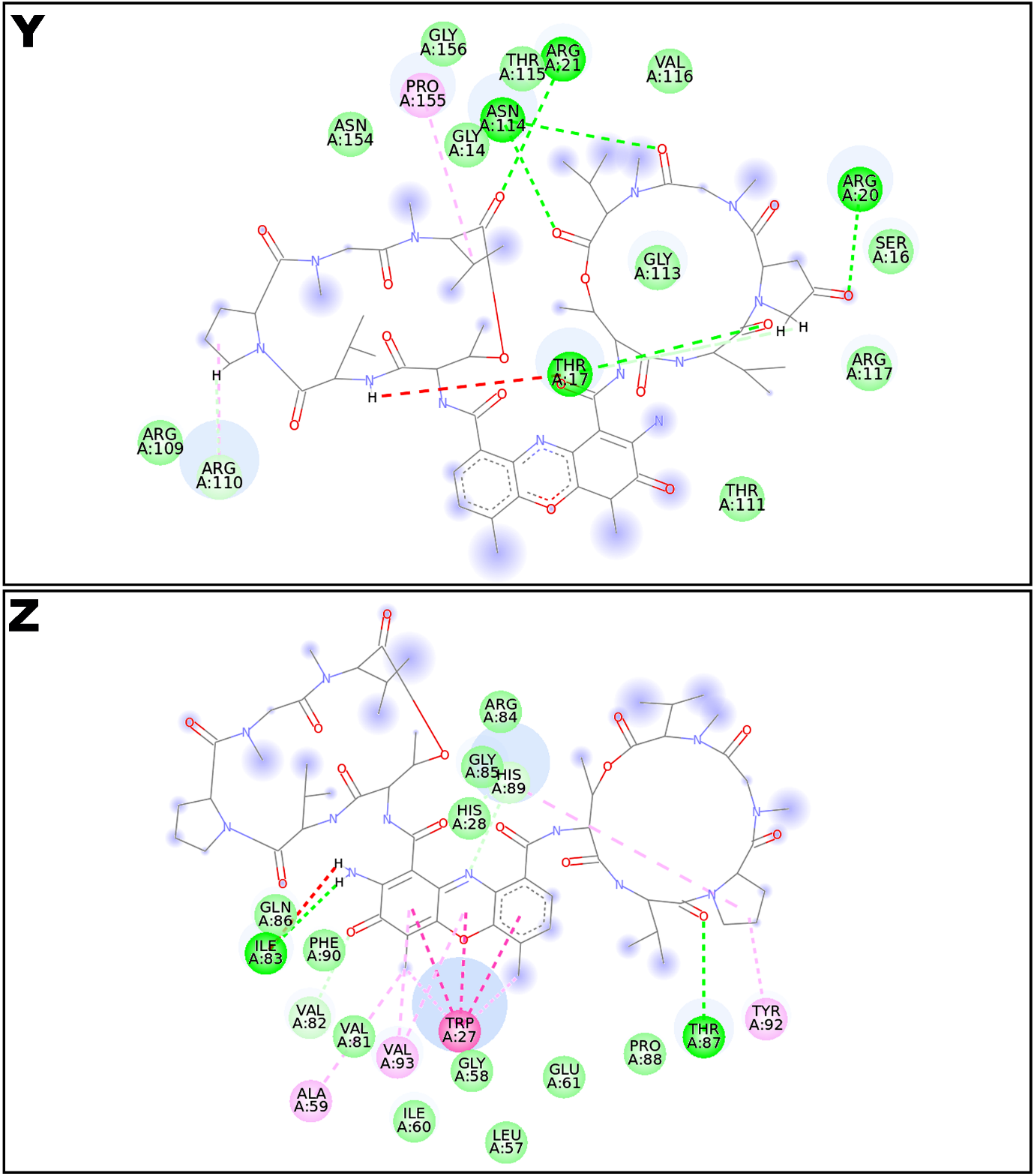


**Figure S17.** Non-bond interactions of act-X_2_ (Y) and act-D (Z) with Mtb shikimate kinase.


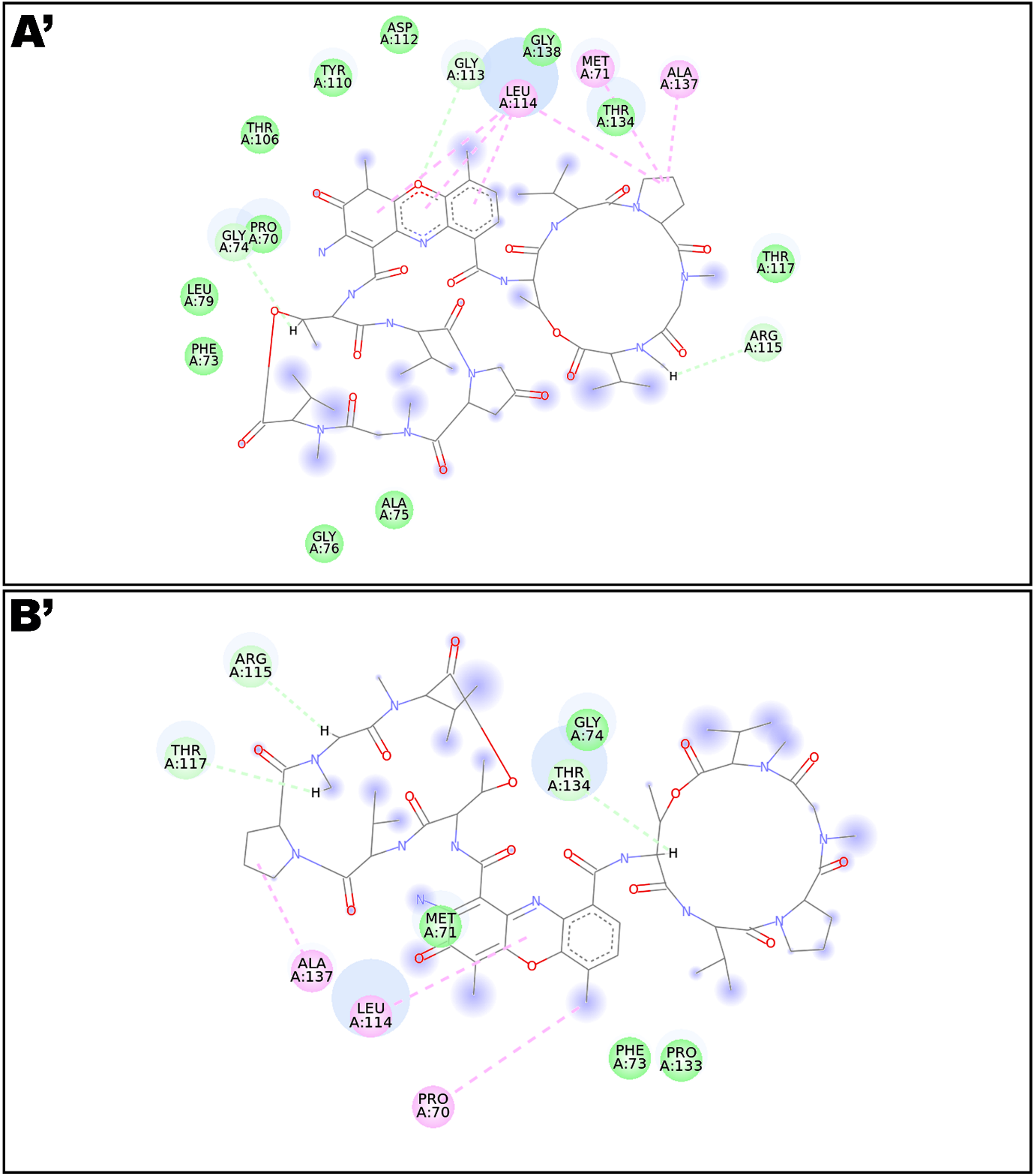


**Figure S18.** Non-bond interactions of act-X_2_ (A’) and act-D (B’) with pantothenate synthetase.


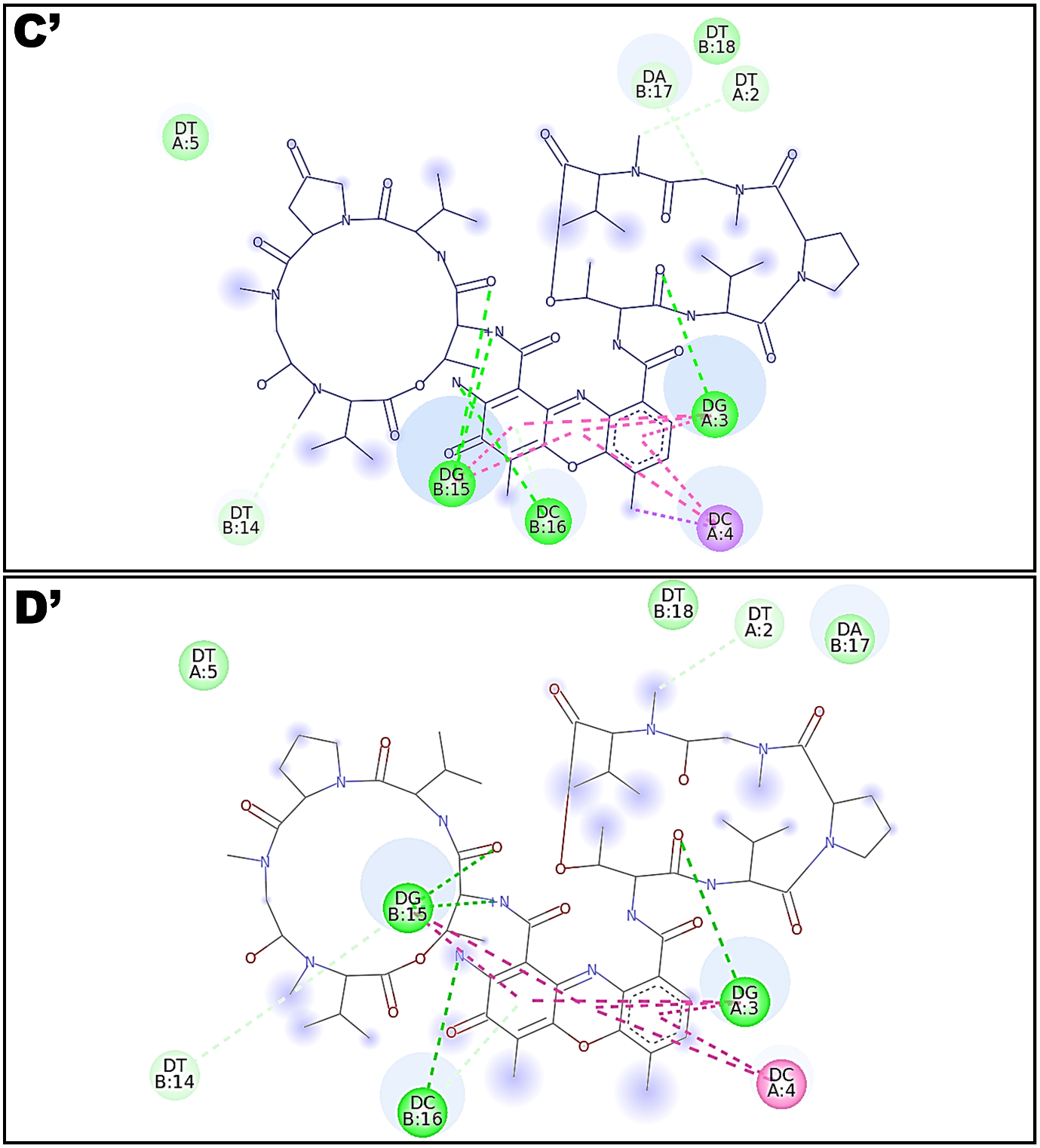


**Figure S19.** Non-bond interactions of act-X_2_ (C’) and act-D (D’) with the DNA.

**Table S1.** The selected proteins for molecular docking studies with act-X_2_ and act-D ligands targeting multiple pathways in *Mycobacterium* strains. The table also lists the name of enzymes, their FASTA sequences, amino acid /nucleotide length, their PDB codes, and the corresponding pathways where the protein is involved.

| **Enzyme** | **Organism** | **FASTA** | **Amino acid/**  **Nucleotide sequence** | **PDB** | **Pathway/Function** |
| --- | --- | --- | --- | --- | --- |
| DNA | *Streptomyces antibioticus* | ATGCTGCAT | 9 | 1MNV | Replication and transcription |
| Protein kinase PknB | Mtb H37Rv | MGSSHHHHHHSSGLVPRGSHMTTPSHLSDRYELGEILGFGGMSEVHLARDLRLHRDVAVKVLRADLARDPSFYLRFRREAQNAAALNHPAIVAVYDTGEAETPAGPLPYIVMEYVDGVTLRDIVHTEGPMTPKRAIEVIADACQALNFSHQNGIIHRDVKPANIMISATNAVKVMDFGIARAIADSGNSVTQTAAVIGTAQYLSPEQARGDSVDARSDVYSLGCVLYEVLTGEPPFTGDSPVSVAYQHVREDPIPPSARHEGLSADLDAVVLKALAKNPENRYQTAAEMRADLVRVHNG | 299 | 2FUM | Biosynthesis and trafficking of mycolic acids |
| Polyketide synthase 13 | Mtb | SNAQIDGFVRTLRARPEAGGKVPVFVFHPAGGSTVVYEPLLGRLPADTPMYGFERVEGSIEERAQQYVPKLIEMQGDGPYVLVGWSLGGVLAYACAIGLRRLGKDVRFVGLIDAVRAGEEIPQTKEEIRKRWDRYAAFAEKTFNVTIPAIPYEQLEELDDEGQVRFVLDAVSQSGVQIPAGIIEHQRTSYLDNRAIDTAQIQPYDGHVTLYMADRYHDDAIMFEPRYAVRQPDGGWGEYVSDLEVVPIGGEHIQAIDEPIIAKVGEHMSRALGQIEADRTSEVGKQ | 286 | 5V3X | Mycolic acid biosynthesis |
| Lumazine synthase | Mtb | MKGGAGVPDLPSLDASGVRLAIVASSWHGKICDALLDGARKVAAGCGLDDPTVVRVLGAIEIPVVAQELARNHDAVVALGVVIRGQTPHFDYVCDAVTQGLTRVSLDSSTPIANGVLTTNTEEQALDRAGLPTSAEDKGAQATVAALATALTLRELRAHS | 160 | 2C92 | Riboflavin biosynthesis |
| Pantothenate kinase | Mtb H37Rv | HHHHHHMSRLSEPSPYVEFDRRQWRALRMSTPLALTEEELVGLRGLGEQIDLLEVEEVYLPLARLIHLQVAARQRLFAATAEFLGEPQQNPDRPVPFIIGVAGSVAVGKSTTARVLQALLARWDHHPRVDLVTTDGFLYPNAELQRRNLMHRKGFPESYNRRALMRFVTSVKSGSDYACAPVYSHLHYDIIPGAEQVVRHPDILILEGLNVLQTGPTLMVSDLFDFSLYVDARIEDIEQWYVSRFLAMRTTAFADPESHFHHYAAFSDSQAVVAAREIWRTINRPNLVENILPTRPRATLVLRKDADHSINRLRLRKL | 318 | 4BFT | Biosynthesis of cofactor CoA |
| Decaprenylphosphoryl-β-D-ribose-2'-oxidase (DprE1) | Mtb CDC1551 | MGSSHHHHHHSSGLVPRGSHMLSVGATTTATRLTGWGRTAPSVANVLRTPDAEMIVKAVARVAESGGGRGAIARGLGRSYGDNAQNGGGLVIDMTPLNTIHSIDADTKLVDIDAGVNLDQLMKAALPFGLWVPVLPGTRQVTVGGAIACDIHGKNHHSAGSFGNHVRSMDLLTADGEIRHLTPTGEDAELFWATVGGNGLTGIIMRATIEMTPTSTAYFIADGDVTASLDETIALHSDGSEARYTYSSAWFDAISAPPKLGRAAVSRGRLATVEQLPAKLRSEPLKFDAPQLLTLPDVFPNGLANKYTFGPIGELWYRKSGTYRGKVQNLTQFYHPLDMFGEWNRAYGPAGFLQYQFVIPTEAVDEFKKIIGVIQASGHYSFLNVFKLFGPRNQAPLSFPIPGWNICVDFPIKDGLGKFVSELDRRVLEFGGRLYTAKDSRTTAETFHAMYPRVDEWISVRRKVDPLRVFASDMARRLELL | 481 | 6HEZ | Synthesis of the cell wall precursor decaprenyl phosphoarabinose (DPA) |
| Protein tyrosine phosphatase PtpB | Mtb H37Rv | MGSSHHHHHHSSGLVPRGSHMAVRELPGAWNFRDVADTATALRPGRLFRSSELSRLDDAGRATLRRLGITDVADLRSSREVARRGPGRVPDGIDVHLLPFPDLADDDADDSAPHETAFKRLLTNDGSNGESGESSQSINDAATRYMTDEYRQFPTRNGAQRALHRVVTLLAAGRPVLTHCFAGKDRTGFVVALVLEAVGLDRDVIVADYLRSNDSVPQLRARISEMIQQRFDTELAPEVVTFTKARLSDGVLGVRAEYLAAARQTIDETYGSLGGYLRDAGISQATVNRMRGVLLG | 296 | 2OZ5 | Tyrosine phosphorylation |
| DNA GyrB ATPase domain | *M. smegmatis* | MPKEYGADSITILEGLEAVRKRPGMYIGSTGERGLHHLIWEVVDNAVDEAMAGFATRVDVKIHADGSVEVRDDGRGIPVEMHATGMPTIDVVMTQVGVSVVNALSTRLEATVLRDGYEWFQYYDRSVPGKLKQGGETKETGTTIRFWADPEIFETTDYNFETVARRLQEMAFLNKGLTIELTDERDGKHRVFHYPG | 196 | 4B6C | Negatively supercoiling of closed circular dsDNA |
| Enoyl reductase | Mtb H37Rv | MTGLLDGKRILVSGIITDSSIAFHIARVAQEQGAQLVLTGFDRLRLIQRITDRLPAKAPLLELDVQNEEHLASLAGRVTEAIGAGNKLDGVVHSIGFMPQTGMGINPFFDAPYADVSKGIHISAYSYASMAKALLPIMNPGGSIVGMDFDPSRAMPAYNWMTVAKSALESVNRFVAREAGKYGVRSNLVAAGPIRTLAMSAIVGGALGEEAGAQIQLLEEGWDQRAPIGWNMKDATPVAKTVCALLSDWLPATTGDIIYADGGAHTQLL | 269 | 4U0J | Fatty acid biosynthesis |
| DNA topoisomerase-I | Mtb H37Rv | SNAADPKTKGRGSGGNGSGRRLVIVESPTKARKLASYLGSGYIVESSRGHIRDLPRAASDVPAKYKSQPWARLGVNVDADFEPLYIISPEKRSTVSELRGLLKDVDELYLATDGDREGEAIAWHLLETLKPRIPVKRMVFHEITEPAIRAAAEHPRDLDIDLVDAQETRRILDRLYGYEVSPVLWKKVAPKLSAGRVQSVATRIIVARERDRMAFRSAAYWDILAKLDASVSDPDAAPPTFSARLTAVAGRRVATGRDFDSLGTLRKGDEVIVLDEGSATALAAGLDGTQLTVASAEEKPYARRPYPPFMTSTLQQEASRKLRFSAERTMSIAQRLYENGYITYMRTDSTTLSESAINAARTQARQLYGDEYVAPAPRQYTRKVKNAQEAHEAIRPAGETFATPDAVRRELDGPNIDDFRLYELIWQRTVASQMADARGMTLSLRITGMSGHQEVVFSATGRTLTFPGFLKAYVETVDELVGGEADDAERRLPHLTPGQRLDIVELTPDGHATNPPARYTEASLVKALEELGIGRPSTYSSIIKTIQDRGYVHKKGSALVPSWVAFAVTGLLEQHFGRLVDYDFTAAMEDELDEIAAGNERRTNWLNNFYFGGDHGVPDSVARSGGLKKLVGINLEGIDAREVNSIKLFDDTHGRPIYVRVGKNGPYLERLVAGDTGEPTPQRANLSDSITPDELTLQVAEELFAT | 706 | 5D5H | To manage the topological state of the DNA |
| NAD^+^-dependent DNA ligase A | Mtb H37Rv | MSSPDADQTAPEVLRQWQALAEEVREHQFRYYVRDAPIISDAEFDELLRRLEALEEQHPELRTPDSPTQLVGGAGFATDFEPVDHLERMLSLDNAFTADELAAWAGRIHAEVGDAAHYLCELKIDGVALSLVYREGRLTRASTRGDGRTGEDVTLNARTIADVPERLTPGDDYPVPEVLEVRGEVFFRLDDFQALNASLVEEGKAPFANPRNSAAGSLRQKDPAVTARRRLRMICHGLGHVEGFRPATLHQAYLALRAWGLPVSEHTTLATDLAGVRERIDYWGEHRHEVDHEIDGVVVKVDEVALQRRLGSTSRAPRWAIAYKYPPEHHHHHH | 334 | 6KJM | Joining the breaks in dsDNA during DNA replication, repair and recombination, utilizing either NAD+ as a cofactor |
| β-ketoacyl synthase-A (KasA) | Mtb | MSQPSTANGGFPSVVVTAVTATTSISPDIESTWKGLLAGESGIHALEDEFVTKWDLAVKIGGHLKDPVDSHMGRLDMRRMSYVQRMGKLLGGQLWESAGSPEVDPDRFAVVVGTGLGGAERIVESYDLMNAGGPRKVSPLAVQMIMPNGAAAVIGLQLGARAGVMTPVSACSSGSEAIAHAWRQIVMGDADVAVCGGVEGPIEALPIAAFSMMRAMSTRNDEPERASRPFDKDRDGFVFGEAGALMLIETEEHAKARGAKPLARLLGAGITSDAFHMVAPAADGVRAGRAMTRSLELAGLSPADIDHVNAHGTATPIGDAAEANAIRVAGCDQAAVYAPKSALGHSIGAVGALESVLTVLTLRDGVIPPTLNYETPDPEIDLDVVAGEPRYGDYRYAVNNSFGFGGHNVALAFGRY | 416 | 2WGE | Biosynthesis of long-chain fatty acids |
| Mtb type II dehydroquinase | Mtb | SELIVNVINGPNLGRLGRREPAVYGGTTHDELVALIEREAAELGLKAVVRQSDSEAQLLDWIHQAADAAEPVILNAGGLTHTSVALRDACAELSAPLIEVHISNVHAREEFRRHSYLSPIATGVIVGLGIQGYLLALRYLAEHVGT | 146 | 2Y71 | Shikimate biosynthesis pathway |
| Diacylglycerol acyltransferase/mycolyltransferase Ag85C | Mtb | MFSRPGLPVEYLQVPSASMGRDIKVQFQGGGPHAVYLLDGLRAQDDYNGWDINTPAFEEYYQSGLSVIMPVGGQSSFYTDWYQPSQSNGQNYTYKWETFLTREMPAWLQANKGVSPTGNAAVGLSMSGGSALILAAYYPQQFPYAASLSGFLNPSEGWWPTLIGLAMNDSGGYNANSMWGPSSDPAWKRNDPMVQIPRLVANNTRIWVYCGNGTPSDLGGDNIPAKFLEGLTLRTNQTFRDTYAADGGRNGVFNFPPNGTHSWPYWNEQLVAMKADIQHVLNGATPPAAPAAPAALEHHHHHH | 303 | 5KWI | Biosynthesis of cell wall |
| Mtb shikimate kinase | Mtb H37Rv | MAPKAVLVGLPGSGKSTIGRRLAKALGVGLLDTDVAIEQRTGRSIADIFATDGEQEFRRIEEDVVRAALADHDGVLSLGGGAVTSPGVRAALAGHTVVYLEISAAEGVRRTGGNTVRPLLAGPDRAEKYRALMAKRAPLYRRVATMRVDTNRRNPGAVVRHILSRLQVPSPSEAATLEHHHHHH | 184 | 2IYQ | Shikimate biosynthesis pathway |
| Pantothenate synthetase | Mtb | MAIPAFHPGELNVYSAPGDVADVSRALRLTGRRVMLVPTMGALHEGHLALVRAAKRVPGSVVVVSIFVNPMQFGAGGDLDAYPRTPDDDLAQLRAEGVEIAFTPTTAAMYPDGLRTTVQPGPLAAELEGGPRPTHFAGVLTVVLKLLQIVRPDRVFFGEKDYQQLVLIRQLVADFNLDVAVVGVPTVREADGLAMSSRNRYLDPAQRAAAVALSAALTAAAHAATAGAQAALDAARAVLDAAPGVAVDYLELRDIGLGPMPLNGSGRLLVAARLGTTRLLDNIAIEIGTFAGTDRPDGYRA | 301 | 3IVX | Pantothenate biosynthesis pathway |

**Note:** Mtb= *Mycobacterium tuberculosis*.

Table S2. *In vitro* anti-TB activity of isolated actinomycins and control drugs.

| Test Compounds | MIC | | |
| --- | --- | --- | --- |
|  | Mtb H37Ra | BCG | Mtb H37Rv |
| Act-X_2_ | 1.56 ± 0.0 µg/mL | 1.56 ± 0.0 µg/mL | 2.64 ± 0.07 µg/mL |
| Act-D | 1.56 ± 0.0 µg/mL | 1.56 ± 0.0 µg/mL | 1.80 ± 0.24 µg/mL |
| S | 6.0 µg/mL | 6.0 µg/mL | NT* |
| RMP | 2.0 µg/mL | 2.0 µg/mL | 0.02 µM |
| INH | NT | NT | 0.46 µM |
| LIZ | NT | NT | 0.92 µM |
| MOX | NT | NT | 0.19 µM |
| PA824 | NT | NT | 0.20 µM |
| TMC207 | NT | NT | 0.01 µM |

**Note:** Act-X_2_=Actinomycin X_2_; Act-D=Actinomycin D; S=Streptomycin; RMP=Rifampin; INH=Isoniazid; LIZ=Linezolid; MOX=Moxifloxacin; PA824=Antituberculosis agent; TMC207= Antituberculosis agent.

*NT= Not Tested

**Table S3.** One-way ANOVA for the in-vitro anti-TB activity of act-X_2_ and act-D.

| **One-way ANOVA** | | | | | |
| --- | --- | --- | --- | --- | --- |
| **Mtb H37Rv** | | | | | |
|  | Sum of Squares | Df | Mean Square | F | Sig. |
| Between Groups | .706 | 1 | .706 | 22.471 | .042 |
| Within Groups | .063 | 2 | .031 |  |  |
| Total | .768 | 3 |  |  |  |
