# Supplementary material for: In vitro and in silico evaluations of actinomycin X2and actinomycin D as potent anti-tuberculosis agents
Source: PeerJ. 2023 Mar 8;11:e14502. doi: 10.7717/peerj.14502 (PMC10022501; doi:10.7717/peerj.14502)
Supplement: Supplemental Information 2 [file peerj-11-14502-s002.docx]

**Raw data for Anti-TB activity of act-X_2_ and act-D**

|  | **MIC of Act-X_2_ for**  ***M. tubercle* H37Rv** | |  |  |  |
| --- | --- | --- | --- | --- | --- |
|  | **Method-1** | **Method-2** |  |  |  |
| **N1** | **2.57** | **2.71** |  |  |  |
| **N2** | **2.62** | **2.68** |  |  |  |
| **N3** | **2.59** | **2.69** | **Mean** | **SD** | **Mean ± SD** |
| **Mean** | **2.59** | **2.69** | **2.64** | **0.07** | **2.64 ± 0.07** |

|  | **MIC of Act-D for**  ***M. tubercle* H37Rv** | |  |  |  |
| --- | --- | --- | --- | --- | --- |
|  | **Method-1** | **Method-2** |  |  |  |
| **N1** | **1.99** | **1.62** |  |  |  |
| **N2** | **1.95** | **1.62** |  |  |  |
| **N3** | **1.97** | **1.65** | **Mean** | **SD** | **Mean ± SD** |
| **Mean** | **1.97** | **1.63** | **1.80** | **0.24** | **1.80 ± 0.07** |

|  | **MIC of Act-X_2_ for** | |
| --- | --- | --- |
|  | ***Mycobacterium bovis* (BCG)** | **Mtb H37Rv** |
| **N1** | **1.56** | **1.56** |
| **N2** | **1.56** | **1.56** |
| **N3** | **1.56** | **1.56** |
| **Mean** | **1.56** | **1.56** |
| **SD** | **0.0** | **0.0** |
| **Mean ± SD** | **1.56 ± 0.0** | **1.56 ± 0.0** |

|  | **MIC of Act-D for** | |
| --- | --- | --- |
|  | ***Mycobacterium bovis* (BCG)** | **Mtb H37Rv** |
| **N1** | **1.56** | **1.56** |
| **N2** | **1.56** | **1.56** |
| **N3** | **1.56** | **1.56** |
| **Mean** | **1.56** | **1.56** |
| **SD** | **0.0** | **0.0** |
| **Mean ± SD** | **1.56 ± 0.0** | **1.56 ± 0.0** |
